# Perineuronal Net Microscopy: From Brain Pathology to Artificial Intelligence

**DOI:** 10.3390/ijms25084227

**Published:** 2024-04-11

**Authors:** Mikhail Paveliev, Anton A. Egorchev, Foat Musin, Nikita Lipachev, Anastasiia Melnikova, Rustem M. Gimadutdinov, Aidar R. Kashipov, Dmitry Molotkov, Dmitry E. Chickrin, Albert V. Aganov

**Affiliations:** 1 Neuroscience Center, University of Helsinki, Haartmaninkatu 8, 00290 Helsinki, Finland; 2 Institute of Computational Mathematics and Information Technologies, Kazan Federal University, Kremlyovskaya 35, Kazan 420008, Tatarstan, Russia; anton@egorchev.ru (A.A.E.); fmmusin@kpfu.ru (F.M.); rumgimadutdinov@stud.kpfu.ru (R.M.G.); 3 Institute of Physics, Kazan Federal University, Kremlyovskaya 16a, Kazan 420008, Tatarstan, Russia; nikita.lipachev@gmail.com (N.L.); alvaraganov@gmail.com (A.V.A.); 4 Institute of Fundamental Medicine and Biology, Kazan Federal University, Karl Marx 74, Kazan 420015, Tatarstan, Russia; anastasiia_melnikova@outlook.com; 5 Institute of Artificial Intelligence, Robotics and Systems Engineering, Kazan Federal University, Kremlyovskaya 18, Kazan 420008, Tatarstan, Russia; ayd.kashipov@gmail.com (A.R.K.); dmitry.kfu@ya.ru (D.E.C.); 6 Biomedicum Imaging Unit, University of Helsinki, Haartmaninkatu 8, 00014 Helsinki, Finland; dmitry.molotkov@embl.it

**Keywords:** perineuronal net, schizophrenia, epilepsy, antidepressant, brain plasticity, machine learning, artificial intelligence, extracellular matrix, synapse

## Abstract

Perineuronal nets (PNN) are a special highly structured type of extracellular matrix encapsulating synapses on large populations of CNS neurons. PNN undergo structural changes in schizophrenia, epilepsy, Alzheimer’s disease, stroke, post-traumatic conditions, and some other brain disorders. The functional role of the PNN microstructure in brain pathologies has remained largely unstudied until recently. Here, we review recent research implicating PNN microstructural changes in schizophrenia and other disorders. We further concentrate on high-resolution studies of the PNN mesh units surrounding synaptic boutons to elucidate fine structural details behind the mutual functional regulation between the ECM and the synaptic terminal. We also review some updates regarding PNN as a potential pharmacological target. Artificial intelligence (AI)-based methods are now arriving as a new tool that may have the potential to grasp the brain’s complexity through a wide range of organization levels—from synaptic molecular events to large scale tissue rearrangements and the whole-brain connectome function. This scope matches exactly the complex role of PNN in brain physiology and pathology processes, and the first AI-assisted PNN microscopy studies have been reported. To that end, we report here on a machine learning-assisted tool for PNN mesh contour tracing.

## 1. Introduction

The human brain can be viewed as a major instrument for the adaptation to environmental conditions. The adaptation takes place via three basic mechanisms:(1) allele frequencies change for species adaptation; (2) epigenetic modifications occur and (3) neuronal networks rewire based on experience for an individual organism’s adaptation. The latter process is commonly termed brain plasticity. Currently, we are witnessing unprecedented progress in research both on the hereditary and ontogenetic mechanisms underlying brain plasticity at various levels, from single synapses to large neuronal networks; perineuronal nets (PNN) can be viewed as an example of this kind [1]. These special structures of the extracellular matrix (ECM) ensheath synapses in large neuronal populations in many regions of the brain and spinal cord, thereby affecting synaptic and neuronal plasticity [2]. Mutations of the genes encoding the PNN components (NCAN, BCAN) are linked to hereditary risks of psychiatric and neurological disorders, so that a correlation of mutations to the severity of behavioral outcomes can be traced in human patients [3].

The PNN has a well-shaped graceful lattice-type structure [4], making it an attractive object for microscopy studies. Indeed, a large amount of structural information can be collected and analyzed quantitatively at a spatial scale, ranging from brain regions to single synaptic terminals. This is quite rare in brain ECMs: in contrast to the PNN, many other types of brain ECMs look poorly structured in microscopy images [5].

The PNN was discovered by Camillo Golgi at the end of the XIXth century, and credit for early studies of brain pathology-related PNN structural changes is also due to Italian neuromorphologists (reviewed in ref. [6]). Further development of biochemistry and molecular biology methods has led to the current view of PNN as a supramolecular complex of chondroitin sulfate proteoglycans (CSPG), tenascin R and link proteins assembled on the hyaluronan backbone [7,8,9,10,11]. This ECM complex serves as a scaffold for a number of extracellular signaling cues, including semaphorin and Orthodenticle homeobox 2 (Otx2), suggesting that the PNN acts as a spatial framework for complex signaling on the neuronal cell surface [12,13,14]. PNN components are synthesized by neurons, astrocytes and oligodendrocytes [10,15]. In particular, the major PNN structural component aggrecan is expressed in neurons and astrocytes. PNN development is triggered by synaptic activity [16,17,18,19,20] and the formation of the PNN terminates the critical period of synaptic plasticity [21,22,23].

A body of experimental evidence points to the pivotal role of the ECM in the function of synaptic networks [21]. As a result, Dityatev and Rusakov proposed the concept of tetrapartite synapse or “synaptic quadriga” [24], highlighting the role of the ECM in synaptic transmission and plasticity. The other three parts of the quadriga, presynapse, postsynapse and astrocytes, have been shown to cross-signal intensely with the surrounding ECM, including the PNN [25,26,27,28]. Therefore, we may expect the ECM to contribute significantly to a range of pathology mechanisms that were previously attributed to neurons and/or glial cells [29]. To date, a body of experimental evidence supports the involvement of PNNs in the pathogenesis of schizophrenia, depression, epilepsy, Alzheimer’s disease, posttraumatic regeneration failure, etc. [4]. Strikingly, PNN research has developed to the point that the PNN is now viewed as a potential pharmacological target [1]. Given the enormous burst in PNN research in recent years, we do not pretend to review all updates on the subject; rather, we hope to grasp some trends that may have a major impact on our understanding of PNN in physiologic and pathologic contexts in the near future. For thorough reviews on the role of PNN in modern neuroscience and biomedicine, please see [1,4,30,31].

Artificial intelligence (AI) tools, including artificial neuronal networks, are becoming more prominent in the research landscape; hence, we are currently facing the prospect of a large-scale conceptual change in brain research in the near future.

Artificial intelligence is the ability of computers to simulate human intelligence in a wide and growing range of tasks.

**Figure 1:**
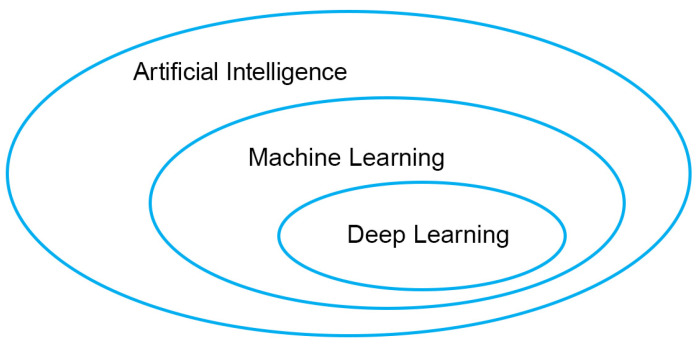
Hierarchy of the machine learning and deep learning methodology domains within artificial intelligence.

Within AI, machine learning (Figure 1) uses statistical algorithms with the initial step of training, i.e., learning “how it should work” using a human-annotated (human-processed) dataset also called “ground truth”. As a result of training, the machine emulates data analysis performed by humans. In other words, the AI model performs generalization: the processing of unseen data based on what it has learned from a previous ground truth. Thus, the machine itself is able to make adjustments to the procedure using the ground truth information and without explicit instructions, in contrast to automated algorithm-based image analysis previously used in biomedicine, where all adjustments were typically made by a human researcher. This self-learning ability positions AI as a highly flexible and time-saving tool that is invaluable in biomedical image analysis.

Within machine learning, artificial neuronal networks (ANN) have become a dominating mathematical apparatus, with the initial idea roughly mimicking biological neuronal nets. Input information is processed by a network of many hubs or “neurons” interconnected by edges. Certain weights are assigned to edges, mimicking the synaptic strength of real neuronal connections. Importantly, weights are adjusted throughout the training process. The adjustment serves as the major mechanism of learning. Deep learning is a type of machine learning utilizing ANNs with two or more hidden layers.

Here, we discuss the ECM-related brain and spinal cord pathology research as the rationale for the rapidly advancing PNN structural studies that are ultimately leading to the development of AI-assisted analysis methods as a potential big methodological shift in brain functional anatomy research. We also focus on the important transition from low-resolution microscopy studies of PNN+ cell density and whole cell PNN intensity to high-resolution studies of individual PNN mesh units at the single-synapse level. Finally, we present an AI-assisted tool for PNN mesh contour annotation and quantitative studies of PNN mesh microstructure.

## 2. Low-Resolution versus High-Resolution Microscopy in PNN Research

**Figure 2:**
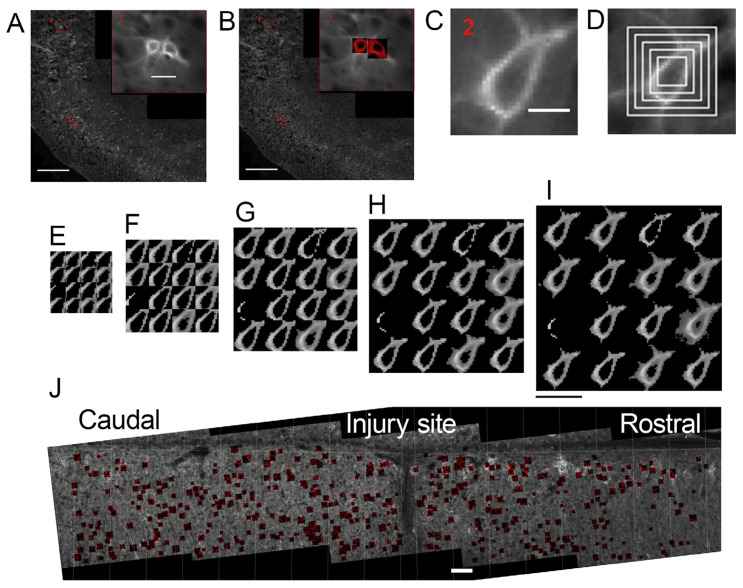
Large-scale tissue-section analysis of the PNN low-resolution microstructure based on epifluorescent microscopy (from [33]): (**A**) WFA-positive PNN in the adult mouse somatosensory cortex, coronal sections, multi-image stitching. The red squares 1 and 2 indicate the areas shown at high magnification in the insert 1 and in (**C**); (**B**) semi-automatic PNN segmentation applied to (**A**). Insert in (**A**,**B**) single cell PNN masks, an example with two neurons; (**C**) a PNN-bearing neuron center is marked manually; (**D**–**I**) five mask size variants—square edge size 10.2; 15.3; 20.4; 25.5; 30.6 μm were applied with 16 autothresholding algorithms; and (**J**) a longitudinal section of the cervical spinal cord after lateral hemisection with single cell PNN masks mapped on it. Scale bar in (**A**) is 500 μm, valid for (**A**,**B**), scale bar in the insert in (**A**) is 25 μm, in (**C**)—10 μm, valid for (**C**,**D**), in (**I**)—25 μm, valid for (**E**–**I**), in (**J**)—100 μm.

PNN microscopy studies can be viewed as two major datasets:

(1) The majority of experimental reports use low optical resolution light (mostly fluorescent) microscopy (10×, 20× objectives, NA within 0.25–0.8 range) to quantify the cell density of PNN+ neurons in tissue sections and to compare the staining intensity of the PNN-associated markers between experimental conditions (Table 1). This is a very important type of methodology and much of our knowledge about PNN structure and function was gained with the help of these techniques (reviewed in [4,10,22]). A number of image analysis tools were developed for the quantification of PNN parameters in low-resolution images [32,33,34,35] (Figure 2). Recently, Lupori and co-authors published “A comprehensive atlas of perineuronal net distribution and colocalization with parvalbumin in the adult mouse brain” [36], raising PNN microscopy studies to a remarkable new level and suggesting new opportunities for high-content structural and functional studies of the brain ECM, as discussed below (review Section 10). Essentially, in this case, machine learning generated a large amount of PNN+ cell annotation data that was spatially resolved and could be transferred to standardized brain atlas coordinates. Thus, among other interesting options, the approach allows for a systematic comparison of the PNN distribution to brain connectomics and spatial transcriptomics data [37].
(2) A smaller number of reports addressed high-resolution structure of single PNN meshes (or single PNN units)—polygonal or round barriers consisting of ECM molecules and surrounding individual synapses [14,20,38,39,40,41,42,43,44] (Table 1). These studies revealed another level of the PNN microstructure, shedding light on the delicate architecture of single synapses and their ECM coat at the sub-micrometer scale. Confocal microscopy was performed with NA = 1.4, allowing for a higher optical resolution [14,20,42]; higher resolutions were obtained with Superresolution Structured Illumination Microscopy (SR-SIM) (Zeiss, Oberkochen, Germany), stimulated emission depletion (STED), stochastic optical reconstruction microscopy (STORM) (Nikon, Tokyo, Japan), AiryScan (Zeiss, Oberkochen, Germany) super-resolution [39,40,44] and electron microscopy (Zeiss, Oberkochen, Germany) [38,43].

As the two datasets capture different features of the PNN microstructure (brain tissue and whole cell level at a low resolution versus the single synapse level of detail at a high resolution), we first review the larger corpus of scientific reports (low-resolution microscopy) in connection with PNN normal function and pathologic implications. We then discuss possible effects of the PNN mesh geometry on synaptic function and, finally, we review single-mesh/single synapse high-resolution PNN microscopy studies.

**Table 1:** A list of PNN microscopy studies sorted according to the imaging technique and disease/or experimental manipulation. The objective lens/numerical aperture values are shown next to the reference numbers for those reports where it could be found.

Method	Disease (Model) or Manipulation	References	Markers
Non-fluorescent light microscopy	Normal brain and spinal cord	[9,45,46,47,48]; [49] (×40); [50,51]	Neurocan, Cat-301, versican, phosphacan, WFA, PV, HABP, TN-R, aggrecan, Sema3A, Sema3B, neurocan, brevican, Crtl1, NG2, APC, GFAP, NeuN, HAPLN1, CD44, BRAL2
	Alzheimer’s disease (AD)	[52] (×10; ×20)	Wisteria floribunda agglutinin (WFA)
	Schizophrenia	[53]; [54] (×1.6; ×40); [55] (×2.5/0.12; ×20/0.5); [56]	WFA, Aggrecan (Cat 301), CS56, 3B3, GFAP, ACAN
	Crtl1/Hapln1 deficiency	[57]	WFA
	TauP301L—Acan mouse model	[58]	Aggrecan, ChAT
	Dementia	[59,60]	WFA, Cat-316, Sema3A, NeuN
	Sleep deprivation	[61] (×40)	WFA
	Substance use disorder	[62] (×20; ×40)	WFA
	Monocular deprivation	[63]	Cat-315, Crtl-1
	Spinal cord injury	[64]	WFA, 2B6
Epifluorescent microsopy	Normal brain	[65]; [33] (×10/0.6; ×20/0.8); [49] (×40); [36] (×10)	WFA, Kv3.1b, Cat-301, Neurocan, brevican, versican, phosphacan, TN-R, HABP, aggrecan, PV, GFAP
	In vitro modeling of PNNs	[66]	WFA, Has-3, aggrecan, Crtl1
	Sema3A binding to the PNNs	[67]	WFA
	Spinal cord injury	[68]; [33] (×10/0.6; ×20/0.8); [69]	WFA, PV, NeuN, aggrecan, Crtl1, ChAT, HABP
	tPA deficiency in FS-PV interneurons	[70]	WFA, PV, GABA, NeuN, Iba1
	Perinatal penicillin exposure	[71] (×10/0.45)	WFA, PV
	Substance use disorder	[72] (×10; ×20; ×40); [73]	WFA
	Hibernation	[74]	WFA
	Epilepsy	[75] (×10)	WFA, PV, Cat-315
	Schizophrenia	[76] (×20/0.75; ×60/1.4); [77] (×40)	WFA, PV, aggrecan, NeuN, 8-oxo-DG
	AD	[78]	WFA, PV, PCP4
	Neuropilin1-Fc injection to visual cortex	[79] (×20/0.5)	WFA, PV, Sema-3A
	Ptprz1 deficiency	[11] (×10; ×20; ×63)	WFA, aggrecan, HAPLN1, neurocan, brevican, tenascin-R 619, phosphacan
	4-methylumbelliferone treatment	[80] (×20)	WFA
	Purkinje Cell Degeneration	[81] (×10; ×63/1.4)	Aggrecan, GAD 65/67, vGlut1, vGlut2, brevican, Haplnq, Hapln4, HABP, TN-R, GFAP
	Ventral hippocampal PNN depletion	[82]	WFA, PV
	Monocular deprivation	[21] (×20; ×40), [83] (×20); [84,85]	WFA, neurocan, PV
Confocal microsopy	Normal adult brain	[38]; [14] (×5/0.16; ×63/1.40); [32] (×20/0.7); [45] (×20), [86] (×20), [87]; [48] (×40), [88] (×63); [50] (×20), [51]; [89] (×40/1.1); [90] (×40)	WFA, Sema3A, SV2, GAD67, aggrecan, versican, phosphacan, TN-R, PV, NeuN, ChAT, neurocan, brevican, calbindin, C6S, GlyT2, vGlut1, Hapln1, GlycR, GABAaR, substance P, PSD95, Ankyrin G, Cat-315
	Enriched environment	[91] (×100/1.4); [92,93]	WFA, PV, GAD67, Aggrecan, Neurocan, VGlut1, Sema3A, calbindin, VGlut2, SMI32
	Co-culture of hippocampal neurons and cortical astrocytes	[94]	Aggrecan, vGlut, PSD-95, VGAT, gephyrin
	lenti-cmv-Nptx2-myc injection to somatosensory cortex	[95] (×40)	WFA, NeuN, PV
	Eyeblink conditioning	[25] (×63)	WFA, VGAT, gephyrine, NeuN, aggrecan
	AD	[52] (×20); [96] (×20, ×63); [97] (×10/0.3; ×63/1.4); [98]	WFA, Aβ (Amylo-Glo), CD68, Iba1, Thioflavin-S, PV, Aggrecan, Crtl1, GAD65/67, vGlut1, Cat-301, calretinin, MAP2, VGAT, brevican
	Schizophrenia, bipolar disorder	[42] (×20/0.5; ×63/1.4); [99]; [100] (×20/0.5); [101] (×20; ×40)	WFA, PV, HNK-1, S100-β, CS56, MMP9, 8-oxo-dG, CD68, Iba1
	Substance use disorder	[102] (×20/0.7; ×63/1.4); [103] (×20/0.75; ×20/0.7); [104] (×40); [105] (×63/1.4); [106] (×40), [107]; [32] (×20/0.7); [62]	WFA, PV, GAD65/67, VGlut1, c-Fos, Calretinin, mGluR2, SMI32, SYN1
	Amyotrophic lateral sclerosis (ALS)	[108]	WFA, aggrecan, NeuN
	Dementia	[60]	WFA, HAPLN1, 6B4, 7B7 Cat-316, Sema3A
	Epilepsy	[109]; [75] (×10; ×100)	WFA, PV, Cat-315, GFAP
	Huntington’s disease	[96] (×20)	WFA, Iba1, PV
	Cartilage matrix deficiency	[110] (×63)	WFA, aggrecan, GABA, PV, Hapln1, brevican, tenascin R, versican, phosphacan, HABP
	tPA deficiency in FS-PV interneurons	[70] (×40/1.44)	WFA, PV, aggrecan
	Deletion Npy1r in forebrain excitatory neurons	[111] (×40/1)	WFA, aggrecan, PV, c-Fos, NeuN
	Acan gene deletion	[112] (×10; ×63); [113] (×10/0.45; ×63/1.4)	Aggrecan, WFA, Tn-R, versican, neurocan, Ctrl-1, brevican, phosphacan, Bral2, PV
	Brevican gene deletion	[114] (×63/1.2)	Brevican, aggrecan, neurocan, HAPLN1, calbindin, CtBP2, HAPLN4, vGlut3, Cav1.3, CtBP2, GluR2/3, GluR4, MBP, SMI32
	Monocular deprivation	[115] (×10/0.45)	WFA, PV
	Fear conditioning	[116] (×40); [117] (×40/1.4); [118]	WFA, Hapln1, PV, Zif268
	Oxidative stress	[119] (×20; ×40; ×63); [120]	WFA, PV, 8-oxo-dG, calbindin, calretinin, Lipofuscin, SMI 311, CSPG
	Fluoxetine treatment	[121]; [122] (×10/0.45); [123]	WFA, PV
	Anxiety (maternal separation with early weaning)	[124] (×20; ×63/1.4)	WFA, PV, OTX2, SST, CR
	Tetrodoxin, NBQX, diltiazem treatment	[125]	WFA, PV, tenascin-R (monoclonal a/b 596), Aggrecan, HABP, NeuN, Synbrev, GFAP, VGAT
	PLX3397 treatment	[126] (×10/0.3; ×63/1.4)	WFA, PV, versican
	Somatosensory deprivation (whisker shaving model)	[20] (×63/1.4)	WFA, VGAT
	Enriched environment, cartilage LP1 deficiency	[127] (×63)	WFA, SMI32, HABP, calbindin
	Deletion of chondroitin 6-sulfotransferase (chst3)	[128] (×63)	WFA, PV
	Poly I:C injection during gestation	[129]	Aggrecan, vGlut, PSD-95
	Tenascin-C, tenascin-R, brevican, neurocan deficiency	[130] (×63)	Aggrecan, PSD95, vGlut1, VGAT, gephyrin, NF200, WFA
	tenascin-R deletion	[131]	WFA, TN-R, PV, ChAT, aggrecan, NeuN, TN-C
	Early social isolation	[132] (×10)	WFA, PV
	Social disfunction model	[133] (×20; ×63)	WFA, PCP4, OTX2, PV, RGS14
	Unilateral labyrinthectomy	[134] (×63)	WFA, SMI32, NeuN, brevican,
	PNN removal	[135] (×4/0.2; ×60/1.4); [136] (×10/0.4); [137]; [138]	WFA, vGlut1, vGlut2, VGAT, PV, aggrecan, versican, brevican, neurocan, phosphacan, proteoglycan Di-4S (2B6)
	Spinal cord injury	[139] (×40, ×63); [140]	WFA, ChAT, NeuN, β-III Tubulin, 5-HT, Iba1, GFAP, Cat-301
Multiphoton microscopy	Normal brain	[89] (×10/0.6; ×25/0.95)	WFA
Super-resolution microscopy	Ischemia	[39] (×20/0.8; ×100/1.46); [40] (×10/0.45; ×20/0.8; ×100/1.46)	WFA, Iba1, GFAP, PV, Kv3.1, VGAT, VGluT1, aggrecan
	Rett syndrome	[41] (×60/1.4)	WFA, synaptotagmin-2, PV, VGLUT2
	Pain	[44] (×63/1.4)	Aggrecan, Pax2, NeuN, VGAT, VGLUT2, Gephyrin, c-Fos, WFA, CD68, Iba1
Electron microscopy	Normal brain	[38,43]	WFA
	Enriched environment	[91]	WFA
	AD	[98]	Brevican, aggrecan (HAG7D4)
	Hibernation-like state	[113]	
AI-assisted	Normal brain	[34,36]	WFA, parvalbumin

## 3. Normal Brain Functions Addressed with PNN Microscopy

### 3.1. PNN in Fear and Memory

Memory is one of the most intriguing brain functions where PNNs are implicated [30,141]. The chondroitinase ABC (ChABC)-induced degradation of PNNs in the basolateral amygdala renders fear memories susceptible to erasure [116]. Recently, Ramsaran and colleagues demonstrated that the developmental assembly of PNN in the CA1 hippocampus was necessary and sufficient for the formation of sparse engrams and precise memories [142].

The neuropeptide Y receptor Y1R has been shown to affect spatial learning via regulation of the PNN formation in the CA1 hippocampus [111]. Conditional depletion of the Npy1r gene led to an increase in the PNN and c-Fos expression in the dorsal hippocampus CA1 and learning deficits. The ChABC treatment restored normal c-Fos expression and learning behavior. From a methodological perspective, the study is interesting for the quantitative image analysis of the number of 10 μm spaced concentric ring intersections (Sholl analysis) revealing the complexity of the PNN-coated dendrite arborization.

The crucial role of the cartilage link protein Crtl1 in making fear memories resistant to deletion has been shown in the experimental model of fear extinction in Crtl1-KO mice [118]. Crtl1 is upregulated during brain development and participates in PNN condensation [46,143]. Expression of the immediate early gene Zif268 was upregulated in the PNN+ neurons in the amygdala upon fear conditioning and was attenuated after extinction training in Crtl1-KO mice as compared to wild-type control animals [118] illustrating the PNN-dependent mechanism of memory retention.

The physiological role of the chondroitin sulfation patterns within CSPG received substantial attention in PNN functional studies [22,30]. Chondroitin 4-O-sulfation was recently shown to regulate PNN formation in hippocampal CA2 and social memory in mice [144]. Brain-specific deletion of the chondroitin 4-O-sulfotransferase gene Chst11 resulted in upregulation of the expression of PNNs surrounding excitatory CA2 pyramidal neurons, an imbalance of excitatory and inhibitory synapses, and abnormally high second interaction times in the two-trial social memory test. ChABC injection in CA2 of the Chst11cKO mice resulted in restoration of normal second interaction time in the social memory test, suggesting that PNN overexpression was responsible for the behavioral abnormality.

A number of recent studies addressed sex-related differences in PNN expression related to fear and memory. The higher expression of PNNs in the retrosplenial cortex was associated with poor performance.

### 3.2. Metal Binding

The negatively charged chondroitin sulfate chains of PNN components bind metal cations that play an important role in membrane currents, calcium signaling, neurotransmission and brain development [10]. The zinc cations bind to hyaluronic acid and compete against the iron cations, thereby acting as antioxidants [144]. The zinc-binding zinc-2-glycoprotein (ZAG) localizes to PNN on PV+ neurons in the cortex and hippocampus [145]. Molecular docking reveals the interaction mode of GAGs with ZAG and its complex with β3 adrenergic receptor (β3AR). The latter was co-localized with PV interneurons and CA2 pyramidal neurons in the hippocampus. Recombinant ZAG prevented apoptosis in cell cultures, suggesting a possible anti-apoptotic mechanism for the PNN-bound ZAG in vivo. The PNN also bind redox reactive metal cations—iron and copper, thereby providing tight regulation of its local concentrations [144].

## 4. PNN Structural Studies in Brain Pathology

### 4.1. PNN Structural Studies in Schizophrenia

Thorough reviews on PNN research in schizophrenia have been published recently [4,31,146]. Here, we focus on the most recent experimental updates and discuss PNN–schizophrenia research as an example of the methodological transfer from low-resolution to high-resolution PNN microscopy studies.

The prefrontal cortex (PFC) synaptic circuitry is one of the crucial functional nodes affected in the schizophrenia pathogenesis (reviewed in [4,31,146]). Its functioning is tightly controlled by the inhibitory GABAergic synaptic input from the PNN-bearing PV+ interneurons (fast spiking interneurons, FSIN) [147]. The reciprocal GABAergic synapses interconnect FSIN networks and synchronize the excitatory state of large numbers of pyramidal neurons for gamma oscillations that are disrupted in schizophrenia [31]. A number of studies suggest that impairments of FSINs in PFC may be the direct cause of schizophrenia symptoms [148,149,150]. PV+interneuron density is reduced in PFC, as demonstrated by a meta-analysis [151], although some studies reported no reduction [99]. Gene expression profiling demonstrates the dysregulation of gene expression for several ECM proteins in schizophrenia, including the PNN components brevican and neurocan [3].

Matuszko and co-authors used confocal imaging (20×/0.50 objective) of PV+/WFA+ mPFC neurons in a ketamine model of schizophrenia to demonstrate the reduction in PV expression accompanied by a reduction in WFA+ cell density. Notably, the WFA intensity remained unchanged, as demonstrated by an elegant “donut”-shaped region of interest approach [100]. By contrast, a high-resolution confocal imaging study (63×/1.4 objective) by the same group using the same ketamine model revealed profound quantitative changes in the fine microstructure of the PNN mesh units [42], providing an example of PNN structural changes that are grasped by high-resolution but not low-resolution microscopy and that may have important functional implications for schizophrenia pathogenesis. 

Thus, PNN research in schizophrenia presents a striking example of the conceptual shift from low- to high-resolution structural studies when the large accumulated body of experimental evidence on the role of PV+ neurons in the disease pathogenesis and on the role of PNN for the high-frequency firing capacity of those neurons led to the high-resolution studies of the PNN mesh unit geometry [42].

A very recent report integrates the concepts of critical period and oxidative stress, demonstrating a novel mechanism that may be crucial for schizophrenia onset [152]. Zhang and co-authors addressed the functional connection between the peroxisome proliferator-activated receptor PPARγ coactivator-α (PGC-1α) expression in PFC and the plasticity critical period timing. The results obtained with PGC-1α KO mice suggest that the oxidative damage of PNNs disrupts the critical period timing, leading to schizophrenia-like behavioral outcomes. Notably, the study used a combination of low-resolution fluorescent microscopy and a transmission electron microscopy (TEM) experimental setup to reveal the synaptic terminal ultrastructure. The authors demonstrate a reduction in the synaptic number accompanied by a decrease in the PSD width and an increase in the synaptic cleft width. A TEM study with the same experimental setup complemented by PNN staining for electron microscopy [38] would be an important further extension of the method so that the PNN+synapse complex ultrastructure could be addressed.

### 4.2. Epilepsy

Epileptic brain hyperactivity causes the MMP9-induced cleavage of the major PNN CSPG component aggrecan, and the naked hippocampal PV+ interneurons become susceptible to the hyperactivity-induced degeneration [153]. Brain tumors have been shown to release proteolytic enzymes that degrade the PNN [27,154]. This leads to an increase in the PV+ interneuron membrane capacitance and a decrease in the firing rate. In contrast to the previous studies, Ueno and co-authors reported an increase in the WFA- and Cat-315-positive PNNs in the hippocampus of pentylenetetrazol (PTZ)-kindled mice [75].

The Depdc5 gene deletion in the mouse forebrain dorsal progenitors causes PNN loss, resulting in PV+ interneuron degeneration and the onset of epilepsy [109]. DEPDC5 is a common causative gene in patients with epilepsy and malformation of cortical development (MCD) thus suggesting a core role for PNN in the pathogenesis of this particular inherited type of epilepsy. 

### 4.3. Alzheimer’s Disease

A time course analysis of the 5xFAD mouse AD model reveals that PV+ interneuron loss occurs only after PNN degradation, suggesting a causal connection between the two degeneration processes [155]. Postmortem cortical tissue from the middle frontal gyrus of AD human patients exhibits a significant reduction in the PNN, with a highly significant negative correlation between the number of PNNs and dense-core plaques. Microglia depletion experiments in 5xFAD mice reveal that the microglia promotes plaque-associated PNN degradation.

Hippocampal CA2 PNN loss is associated with social memory deficits in the Tg2576 mouse model of AD [78]. Strikingly, a single injection of neuregulin-1 rescued the PNN numbers and social memory, suggesting the possibility of new therapeutic approaches.

### 4.4. Drug Abuse

Slaker and co-authors developed an automated method PIPSQUEAK for PNN intensity quantification in low optical resolution images of brain sections [35], creating a region of interest around each PNN and subtracting the image background with the help of the Rolling Ball Radius function in Fiji. The method is instrumental for the analysis of double- and triple-labelled cells. The method was applied in a series of cocaine abuse studies [102,105,156].

The PFC PNN staining intensity decreased after 1 day of cocaine exposure and increased after 5 days of cocaine exposure, both effects accompanied by a decrease in the number of action potentials in FSINs [102]. The WFA staining intensity measured after 5 days of cocaine administration correlated with locomotor activity on days 2 and 3, suggesting that changes in the PNN+FSINs determine the PFC-driven changes in behavior. 

Cocaine memory reactivation was shown to decrease PV intensity in the PFC PNN+ FSIN while the PNN intensity remained unchanged [105]. The ChABC-dependent digestion of PNNs hampered both the acquisition and reconsolidation of cocaine memories [156].

### 4.5. Spinal Cord Injury

The PNN coating of lumbar motoneurons was attenuated following thoracis contusion in mice [157]. Interestingly, physical exercises restored PNN expression and promoted functional recovery. Physical activity also reduced PNN expression in brainstem sensory nuclei, while the spinal cord injury had no effect on that.

Lipachev and co-authors quantified WFA staining intensity, PNN area and PNN density in laminae 6 and 7 of the cervical spinal cord around the injury site 9 weeks after a lateral hemisection applied at C5 [33]. The authors observed changes in the PNN area, CSPG enrichment and the density of PNN-bearing neurons within 1.8 mm rostrally, and 1.2 mm caudally, from the injury site. The analyzed area (C3–C6) of intermediate grey around the central channel is the site of phrenic afferent projections, suggesting that PNN changes may affect posttraumatic regeneration of the phrenic motor control. The authors developed a semi-automatic tool for the quantification of the single-cell PNN area and intensity (Figure 2) and demonstrated its application on roughly 6000 PNN-bearing neurons in the spinal cord (10×/0.6 objective) and some 1800 neurons in the brain somatosensory cortex.

To summarize, the low-resolution tissue-section imaging allows for relatively fast qualitative inspection and quantitative studies of large numbers of PNN+ cells. The major limitation is the low level of subcellular detail. Deep learning algorithms were previously shown to emulate the retrieval of superresolution data from confocal microscopy datasets [158]. Thus one could expect similar approaches to be developed for low-resolution epifluorescent and confocal data to be used for the emulation of high-resolution microscopy data via AI implementation.

## 5. How Could the PNN Mesh Geometry Affect the Synapse?

### 5.1. The Mesh Area

**Figure 3:**
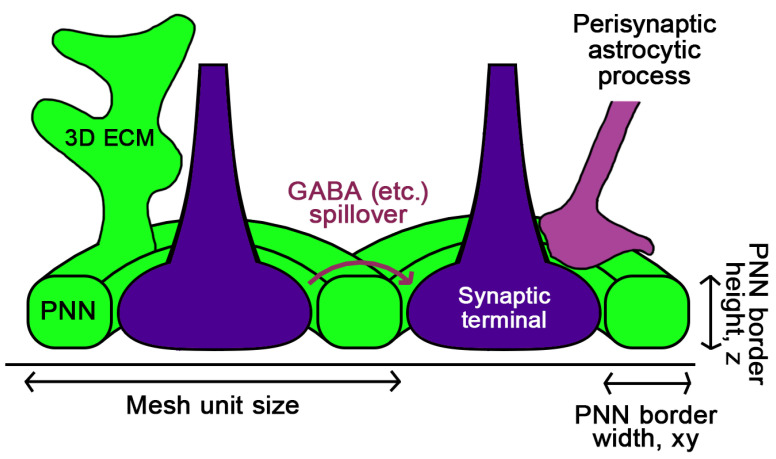
The PNN mesh 3D geometry. The PNN mesh units surround single synaptic boutons and thereby may possibly restrict the synaptic contact area. The mesh border width in xy determines the spacing between synapses. The mesh border width and the mesh border height in z may determine the extracellular space volume and may also affect the spillover of GABA and other signaling molecules released by a synapse. The CSPG-positive 3D ECM represents a continuation of the cell surface PNN layer, spanning over the extracellular space and potentially acting as a scaffold for cells and molecules. PNN and perisynaptic astrocytic processes together form the synapse “coat” controlling local molecular concentrations.

One interesting question about PNN-coated synapses is whether the mesh borders control the size of the synaptic contact and prevent the two cells from increasing (or also decreasing) the synaptic contract area (Figure 3). Here, we use the term “PNN mesh” to designate an ECM border around a single synapse as a structural unit of the net. Three-dimensional EM data suggest that the PNN tightly wraps synapses in deep cerebellar nuclei and the hippocampal CA1 area, restricting the size of the synaptic contact [43]. Changes in the mesh area were reported in an experimental schizophrenia model [42], dark rearing, and in the Rett syndrome experimental model [41].

### 5.2. The Mesh 3D Thickness

The height of the PNN “wall” around the synapse (the mesh 3D thickness) may have a significant impact on synaptic transmission.

First, it may determine the local volume of the extracellular space above the neuronal plasma membrane.

Second, it affects the amount of negatively charged CS around the synapse, i.e., the buffering capacity for cations and positively charged ligands (neurotrophic factors, Otx2, etc.).

Third, it may have a crucial effect on the GABA spillover to neighbouring synapses (Figure 3). Astrocyte current recordings under the ChABC treatment suggest that PNN barriers are required for glutamate and K^+^ uptake by astrocytes and that the ChABC-induced PNN digestion causes the glutamate and K^+^ spillage to the extrasynaptic space [28]. For GABA, the PNN-dependent control of extrasynaptic spillage may also be significant because the GABA concentration around the synaptic cleft is not regulated as tightly as the glutamate concentration via high-affinity uptake [24,159,160]. Besides GABAergic and glutamatergic synapses [20,28,40], other types of synapses have not been shown, to our best knowledge, to populate PNN meshes. Hippocampal PV+ interneurons receive dopaminergic innervation from ventral tegmental area (VTA) and the firing rate was reduced significantly in those neurons upon VTA dopamine neuron degeneration in the TG2576 mouse model of AD [161]. Futhermore, cortical PV+ interneurons exhibit an abnormal PNN structure, altered action potentials, and deficits in dopaminergic modulation in mice carrying a truncated allele disrupted in schizophrenia allele [162]. These data suggest that the PNN of PV+ neurons may harbor dopaminergic synapses of high physiologic and pathological importance, and that the PNN of spinal motoneurons likely contain serotoninergic synapses [163].

### 5.3. The Intersynaptic Layer Width

The X-Y width of the PNN border between synapses (Figure 3) may affect crosstalk between the neighboring synapses both in terms of the spill-over of neurotransmitters and the propagation of postsynaptic currents. PNN was shown to act as the insulator regulating the plasma membrane capacitance of the postsynaptic neuron [27].

There is experimental evidence suggesting that the width of the PNN strands affects the cell surface area available for synaptic contacts and astrocytic coverage. Indeed, PNN removal by ChABC increases the number of inhibitory synapses on excitatory neurons of deep cerebellar nuclei [25] and the number of excitatory synapses on hippocampal neurons [113]. The number of VGAT-negative spaces was strongly decreased under ChABC (confocal microscopy) and the average distance between GABAergic terminals was much lower as compared to the control (EM) [25]. This type of control of synaptic contacts by CSPG ECM is not restricted to cell bodies, as a similar increase in the synaptic contact number was observed in spiny dendrites under ChABC treatment [164]. However, it should be noted that the increase in the GABAergic synapse number was accompanied by a decrease in the glutamatergic synapse number [25].

PNN digestion with ChABC resulted in an increase in the cell surface portion covered with astrocytic processes [28], further suggesting that the width of the cell surface PNN strands restricts the cell–cell contact area.

At present, EM techniques give the most accurate quantitative estimation of the width and height of the PNN layer around synapses and the synaptic terminal area. Broader application of super-resolution imaging techniques and quantitative image analysis may also expand the range of epitope-specific markers and the structural parameters to be quantified.

When addressing the effects of the PNN mesh geometry on synaptic function, one could expect bi-directional regulation between the PNN and synapses. It was previously hypothesized that CSPG enrichment within an individual PNN unit may be affected by the firing activity of the corresponding synapse via the secretion of ECM molecules or, vice versa, via the secretion of ECM-degrading proteases [42].

## 6. PNN Single-Mesh Studies

While significant information was accumulated at a low level of structural detail, the studies of PNN single-mesh morphology, i.e., the geometry of the ECM layer surrounding individual synaptic contacts, remained purely qualitative for a very long time. The most convincing evidence of the WFA-positive extracellular material surrounding individual synaptic boutons was provided by Bruckner and co-authors by means of transmission electron microscopy on large, superior colliculus neurons [38]. Using STORM, Korotchenko and co-authors reported a profile of GAD65 and aggrecan fluorescence on the cell surfaces of cultured hippocampal neurons [165].

**Figure 4:**
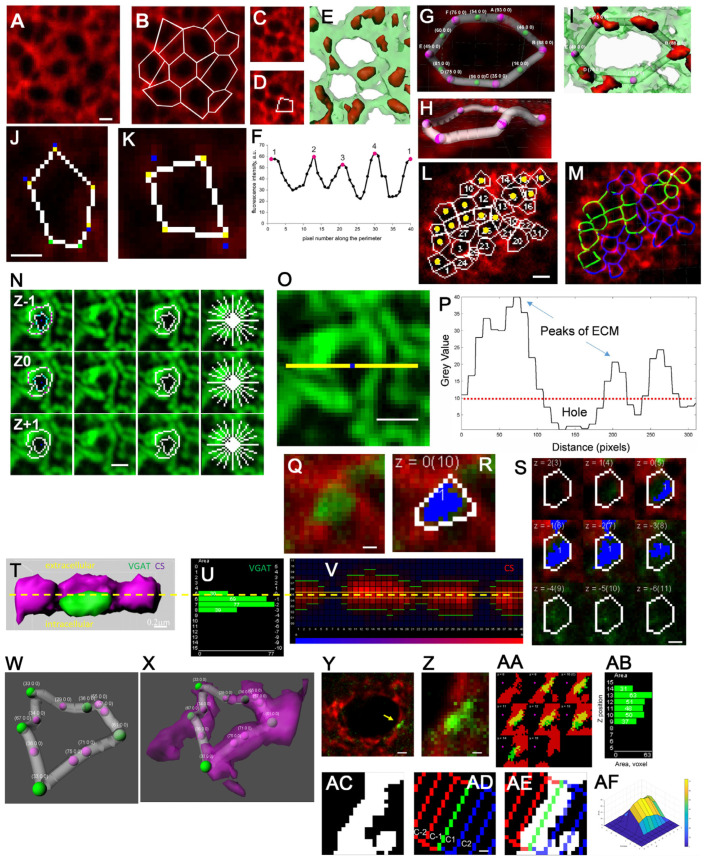
Quantitative analysis of the high-resolution single-mesh PNN microstructure (from [14,20,42]): (**A**,**B**) PNN geometry analyzed by manual tracing of individual meshes approximated as triangle, quadrilateral, pentagon, hexagon, and polygons with a higher number of vertices. Pentagons and hexagons are the most common shapes; (**C**–**F**) the “polar” pattern of chondroitin sulfate distribution along the mesh perimeter; (**C**) a confocal image of the somatosensory cortex neuron showing three meshes with node-enriched (polar) distribution of the WFA-binding epitope; (**D**) the mesh perimeter annotation. Vertices 1–4 are shown; (**E**) 3D reconstruction of chondroitin sulfate distribution for the area shown in (**C**,**D**). An isosurface for moderate fluorescence intensity is shown in green, semitransparent. An isosurface for high fluorescence intensity is shown in red; (**F**) chondroitin sulfate intensity profile along the perimeter of the mesh traced in (**D**). Vertices are shown in purple; (**G**–**I**) three-dimensional reconstruction of the mesh perimeter with filament autodepth tracing. Vertices are shown in purple, middle pixels of each edge are shown in green. Intensity values of the mesh vertices and middle pixels of each edge are shown in (**G**); (**I**) vertices A, B, D, F are surrounded by small volumes of the high-intensity chondroitin sulfate staining. An isosurface for moderate fluorescence intensity is shown in green, semitransparent. An isosurface for the high-intensity threshold is shown in red; (**H**) the “side view” is shown for the same mesh as in (**G**,**I**); (**J**,**K**) local chondroitin sulfate density maxima (blue) exhibit closer match with vertices of polar meshes (yellow) as compared to non-polar meshes; (**L**,**M)**. PNN mesh clusters with node-enriched and uniform distribution of CSPG. (**L)**. PNN on mouse neuron cell body. Meshes with polarity index above 1.5 are marked with yellow dots. Two clusters of polar meshes are separated by a cluster of nonpolar meshes (center, mesh number 10, 12, 13, 19–22, 31). (**M)**. The same area as in (**L**) was used for 3D reconstruction and polarity quantification. Green—polar meshes, blue—nonpolar. Polarity index threshold of 1.5 was used to discriminate between polar and nonpolar meshes in (**L**,**M**). The 2D and 3D types of analysis detected the same clusters. (**N**) the semi-automated algorithm-driven annotation of the PNN holes and perisynaptic ECM in three planes (Z − 1, Z0, Z + 1). The center of the PNN unit (the blue dot) is selected by the user. The global maxima per specific directions are shown as red dots in three z planes. Cyan color pixels are the first pixels above the threshold used for the determination of holes; (**O**,**P**) chondroitin sulfate intensity distribution across a single mesh (along the yellow line). The threshold for the holes segmentation is shown as a dotted red line; (**Q**) a GABAergic synapse stained for VGAT (one large cluster, green) and WFA (red); (**R**) the PNN mesh annotation (white) and the VGAT-positive object segmentation result (blue), the same area as in (**Q**); (**S**–**V**) the confocal stack for the PNN mesh shown in (**S**) was used for 3D reconstruction of the VGAT (green) and WFA (purple) fluorescence; (**T**) computer modeling of a transversal cut of the synaptic terminal and the surrounding PNN; (**U**) VGAT-positive object area (Z distribution) for the synaptic terminal shown in (**T**); (**V**) WFA fluorescence intensity (Z distribution, color-coded blue-red, the color code given in the bottom of the panel) along the perimeter of the mesh. The Z axis of the confocal stack is aligned for (**T**–**V**). Z = 0 is the Z plane with the maximal WFA signal intensity along the mesh perimeter; (**S**) sequential confocal images within a confocal stack, image segmentation is shown for VGAT (blue); (**W**) a PNN mesh, 3D reconstruction with the filament autodepth instrument (Imaris). The mesh vertices are shown in green, the middle pixels of edges are shown in magenta, corresponding values of fluorescence intensity are given in brackets. (**X**) The 3D surface reconstruction of the WFA staining fluorescence intensity combined with the same filament reconstruction as in (**W**); (**Y**–**AF**) transverse section analysis for the confocal stack of a VGAT-positive synapse; (**Y**) a PNN-coated neuron in the barrel cortex layer IV. A transverse confocal section of the PNN-coated cell surface (red for WFA). The GABAergic synapse (green for VGAT) (arrow) was analyzed in further detail in (**Z**–**AF**); (**Z**) the same synapse + PNN complex as in (**Y**); (**AA**) sequential confocal images segmented for VGAT (shown in green) and WFA (shown in red) within a confocal stack for the synapse shown in (**Z**). The overlay of the VGAT- and WFA-positive object masks is shown in yellow. The intracellular side is marked with a magenta dot; (**AB**) the Z distribution of the segmented VGAT-positive object area for the synaptic terminal shown in (**Z**); (**AC**) a binarized mask for the WFA-positive object in the synapse shown in (**Z**); (**AD**,**AE**) segmentation of the image in (**Z**) into contours based on the distance from the central line of the PNN layer (shown in green) towards the extracellular (blue contours) and intracellular (red contours) space; and (**AF**) the WFA-positive object area distribution in the Z-contour coordinate space for the synapse shown in (**Z**). The scale bar in (**A**) is 0.5 μm, valid for (**A**,**B**), (**J**)—0.5 μm, (**L**)—1 μm, (**N**)—1 μm, (**O**)—1 μm, (**Q**)—0.3 μm, (**S**)—0.5 μm, (**Y**)—1 μm, (**Z**)—0.3 μm, (**AD**)—0.2 μm.

The quantitative spatial structure of PNN mesh units was described by Arnst and co-authors using high-resolution confocal microscopy on the WFA-stained cortical neurons of mice and rats [14]. The authors proposed a PNN geometry annotation method, where a single PNN unit was approximated as a polygon traced with the PointPicker tool in FIJI open source software (Figure 4A,B). The study reported a remarkably high variation in the unit area within the same neuron and demonstrated the pentagon shape to be the most frequent shape variant for the manual polygon tracing method. The polygon method proposed in that study proved to be useful in revealing structural changes within the PNN units in a subsequent study of the experimental schizophrenia model [42].

One unexpected finding of the study was the discovery of the mesh clusters on the neuronal cell bodies based on the CS distribution patterns along the mesh contour [14] (Figure 4C–M).

The effect of the PNN on synaptic structure was addressed with super-resolution STED microscopy followed by quantitative image analysis on hippocampal neurons co-cultured with astrocytes [166]. The authors compared the postsynaptic scaffolds composition between the neurons coated with PNNs and those devoid of them, and revealed a correlation between the PNN expression and the density of gephyrin- and VGAT-positive puncta.

The first detailed super-resolution structured illumination microscopy-derived quantitative description of the PNN microstructure was reported by Dzyubenko and co-authors [39]. The authors developed a graph construction approach to demonstrate PNN topology changes in WFA- and aggrecan-labelled PNN in mouse brain hypoperfusion and focal cerebral ischemia models. Based on their experimental results, the authors proposed the hypothesis of a reversible topological tension regime of the PNN ultrastructure that would be potentially capable of facilitating local rewiring after stroke.

The authors further expanded the PNN topology analysis, addressing simultaneous structural changes in the PNN and presynaptic components of the PNN+synapse complex [40]. Combining STED, SR-SIM and confocal microscopy the authors demonstrated that coherent remodeling of PNNs and their perforating inhibitory synapses was affected by the severity of the ischemic injury. Contributing to the high-resolution connectome view of the synaptic circuitry, the authors quantified that a PNN+ motor cortex interneuron received, on average, 75 GABAergic synaptic inputs, this number increased transiently after a stroke and then decreased by day 42. Furthermore, the authors undertook a comparative test of four high-resolution microscopy methods: multiphoton, confocal, SLIM and STED microscopy on the same PNN samples and demonstrated that the graph analysis was applicable to SLIM and STED but not to multiphoton or confocal data.

Sigal and co-authors used principal component analysis (PCA) to study the postnatal development of PNN and pathology-related changes in a Rett syndrome transgenic model [41]. The authors combined STORM super-resolution microscopy with serial-section reconstruction to demonstrate distinct developmental trajectories and remarkable pathology-associated changes in the PNN high-resolution structure. The mean hole size of the visual cortex PNN was affected by dark rearing, indicating the requirement of a sensory input for proper PNN mesh geometry formation during brain development.

PNN mesh geometry was further addressed in the ketamine model of schizophrenia [42]. The authors developed a semi-automatic method for PNN mesh contour tracing for the mesh geometry quantification both in 2D confocal images and 3D stacks (Figure 4N–P). The PFC PV+interneurons from control and ketamine-treated rats exhibited significant differences in PNN mesh number, area, solidity, and circularity.

The whisker-shaving model of somatosensory deprivation during early postnatal development revealed malformation of the PNN+synapse 3D structure [20] in GABAergic synapses stained for WFA+VGAT and visualized with high-resolution confocal microscopy (Figure 4Q–AF). The PNN mesh 3D structure was more flattened and the VGAT clusters were smaller as a result of the deprivation.

The microglia-dependent PNN degradation in the lamina I spinoparabrachial projection neurons resulted in the excitation/inhibition balance shift. leading to pain behavior after peripheral nerve injury [44]. The authors used super-resolution AIRYSCAN microscopy to show that the number of GABAergic and glutamatergic synaptic boutons remained unchanged upon the peripheral nerve injury and ChABC treatment. The peripheral nerve injury-induced pain was explained by decreased frequencies of the miniature inhibitory postsynaptic currents caused by PNN CSPG degradation.

Tewari and co-authors proposed a very promising PNN image analysis approach based on intensity profile tracing along the cell surfaces in the neuron transverse confocal sections [167]. The method allows for quantitative estimation of PNN integrity and PNN mesh size. The authors then used this type of image analysis to study the complex of the PNN with synapses and perisynaptic astrocytic processes [28]. Interestingly, the intensity profiles demonstrated a profound difference in the PNN-astrocytic markers co-localization between layers 3–4 of the somatosensory cortex and CA2 of the hippocampus, suggesting existence of distinct brain region-specific variants of the tetrapartite synapse structure. Within hippocampal CA2, there was also a remarkable difference in PNN-astrocyte co-localization between stratum pyramidale and stratum radiatum. The functional meaning of those structural differences would be the next exciting question to investigate. Roger Tsien put forward the hypothesis that the PNN may serve as a physical substrate for long-lasting memory storage and proposed a broad methodological perspective for addressing that possibility [141]. Two recent reports addressed the hypothesis with different experimental approaches, including two modifications of volume EM.

Focused ion beam scanning electron microscopy (FIB-SEM) was used on the hibernation-like state (HLS) model to test whether the hippocampal CA1 PNNs store the memory traces that could be restored after the end of HLS and synaptic reconnection [113]. Using ChABC and aggrecan KO, the authors demonstrate that the CA1 PNNs are not required for long-term memory storage.

Another study used serial block face SEM to reveal the 3D ultrastructure of the PNN+synapse complexes [43]. Essentially, all of the surface (more than 98%) of the dendrite plasma membrane was in contact with either PNN or presynaptic boutons in the PNN+synapse example described in the study.

## 7. Perineuronal Net as a Potential Drug Target

The rapid progress of PNN studies in a range of brain disease models highlights the prospects of PNN pharmacological targeting as a new medication approach [62,144,168,169]. Microscopy techniques may be highly instrumental in addressing PNN medical pharmacology both in terms of high-throughput drug lead screening and for the high-resolution investigation of drug targeting and effects at a single-synapse level.

PNN disruption was suggested as a potential therapeutic approach to reactivate brain plasticity in children and adults with autism spectrum disorders (ASD), making those patients susceptible to socialization [168]. The chondroitinase ABC (ChABC)-mediated digestion of CNS CSPG has also been considered as a promising therapeutic approach for improving posttraumatic regeneration in the brain and spinal cord [1,64]. PNN digestion with ChABC improved memory outcomes in a mouse tauopathy model [59], with another effective approach being the injection of antibodies targeting the chondroitin 4-sulfate, attenuating PNN formation and Sema3A binding [60].

The ChABC-induced digestion of CSPG can be further potentiated by lithium administered via intraperitoneal injections of LiCl, as demonstrated in a rat model of spinal cord injury [170]. Lithium may act via bisphosphate nucleotidase 2 (BPNT-2), regulating chondroitin sulfation patterns in the brain [4,171].

CSPG digestion with ChABC results in the large-scale removal of chondroitin sulfates in the brain or spinal cord tissue, which may be viewed as a relatively nonspecific effect in terms of the many possible consequences resulting from the CSPG function disruption. Lentiviral and/or adenoassociated viral vectors for genetically regulated targeted ChABC expression [172,173] might be viewed as a potential tool for improving the targeting specificity.

PNN may be also a potential target for antidepressant pharmacology. It was suggested that PNN could serve as a biomarker (or a readout) in experimental models of depression for testing the efficiency of antidepressants [174]. The selective serotonin re-uptake inhibitor and antidepressant fluoxetine causes a reduction in the PNN coating on the PFC interneurons [121]. Transcriptomics analysis in PV+ interneurons indicates that fluoxetine down-regulates enzymes involved in PNN formation and affects expression of the BDNF/TrkB signaling pathway components [123]. Notably, BDNF, NT-3, GDNF, HB-GAM (pleiotrophin), FGF, VEGF and some other neurotrophic factors have positively charged sites that bind CSPGs with high affinity (please see [14] for references). GDNF and its homolog neurturin overcome the inhibitory action of aggrecan on neurite outgrowth in cultured hippocampal and cortical neurons (Paveliev, Rauvala and Saarma, unpublished). GDNF was tested in clinical trials as a drug lead for anti-Parkinson therapy and could be possibly considered for targeting PNN in ASD, Alzheimer’s disease and posttraumatic regeneration. Another strong CSPG binder, HB-GAM, abrogates the binding of chondroitin sulfates to the receptor phosphatase sigma (PTPRS), promotes dendritic and axonal growth in the injured brain and spinal cord parenchyma, and improves functional regeneration after spinal cord injury [175,176,177]. The sperm-derived peptide protamine binds negatively charged epitopes in chondroitin sulfates and heparan sulfates, and is routinely used in heart and pulmonary surgery as a heparin antidote. A 14aa fragment of protamine called low-molecular-weight protamine (LMWP) prevents PTPRS binding to chondroitin sulfates and promotes posttraumatic functional recovery after spinal cord injury in mice [178]. The binding of HB-GAM and LMWP to the chondroitin sulfate moieties of PNN could partially explain the posttraumatic regeneration-promoting effect, as PNN was previously implicated in the inhibition of axonal regeneration after spinal cord injury [179,180].

The massive negative charges of the PNN CS suggest that PNN may affect the accessibility of synaptic pharmacological targets for negatively charged drug molecules. Short soluble polysialic acid fragments have been shown to inhibit the opening of GluN1/GluN2B channels in vitro and to rescue cognitive deficits in two models of Alzheimer’s disease [181]. This result raises the question as to whether polysialic acid fragments and other negatively charged pharmacological agents can penetrate to their targets located in and next to the synaptic cleft in synapses covered by PNNs. 

## 8. Future Methodological Perspective for PNN Microscopy

### 8.1. Multiphoton Microscopy

Live brain imaging of the PNN structural and functional dynamics has remained absent until very recently, although longitudinal in vivo imaging techniques for brain cells and some ECM components have been around for a while [175,182,183]. To that end, a highly promising methodology-oriented study has reported longitudinal in vivo imaging of the PNN using live brain two-photon microscopy on a mouse barrel cortex [89]. The PNN was stained by intracranial injection of fluorescent WFA. The authors demonstrated the pathology-related reduction in live brain PNN density in a mouse model of fragile X syndrome. They were able to combine live brain PNN and Ca^2+^ imaging and reported different statistical distribution of Ca^2+^ fluxes in PV+ neurons with vs. without PNN. Importantly, PNN degradation by the metalloproteinase ADAMTS4 was also demonstrated with two-photon microscopy in brain slices. Further development of the method would allow for imaging of the PNN in the hippocampus, amygdala and in a range of subcortical structures by using cannulas or prisms implanted with cranial imaging windows [184].

A knock-in transgenic mouse expressing the link protein HAPL1 fused to Venus has been generated via Crispr/Cas9 genome editing [90]. The construct is expressed in the brain, exhibiting a PNN-like structure on the neuronal surface, as visualized by confocal microscopy. This may be very instrumental for multiphoton studies of live brain PNN dynamics in a range of pathology models.

### 8.2. Super-Resolution Microscopy

A deep learning algorithm based on training a generative adversarial network (GAN) to transform diffraction-limited input images into super-resolved ones has been reported [158]. The method was shown to transform confocal images to match the resolution characteristics of STED microscopy. Another cross-modality conversion allowed for transformation of total internal reflection (TIRF) images to match the results obtained by TIRF-based structured illumination microscopy. Deep learning approaches have also been shown to increase dramatically the super-resolution microscopy throughput for single molecule localization in the nuclear pore and in mitochondria imaging applications [185]. These approaches can be expected to push PNN microscopy towards high-content super-resolution data acquisition and/or processing for large-scale tissue and brain connectome studies.

### 8.3. Electron Microscopy

The rapid development of EM 3D imaging and reconstruction methods suggests exciting perspectives for PNN ultrastructural studies. Indeed, volume EM techniques including array tomography and serial section TEM offer experimental protocols for 3D EM imaging of brain tissue [186,187]. Notably, a validated set of antibodies for array tomography-based imaging of brain synapses has been reported [188]. The attractiveness of the high xy resolution offered by array tomography is counterbalanced by its low z resolution. The serial block face SEM modification of volume EM may be especially attractive [189] as it allows for a large imaging volume (some 100 × 100 micrometers) along with a high 3D resolution. An even higher resolution (some 4 × 4 nm voxel size) is characteristic of focused ion beam volume EM modification [190], which is counterbalanced by a smaller imaging volume size as compared to the serial block face.

The next crucial step after the EM data collection is image analysis and 3D modeling [191]. Machine learning tools are advancing enormously in these applications [192], suggesting that detailed 3D models of the PNN mesh + synaptic terminal complex will arrive in the near future at resolutions of 10–50 nm. Volume EM is currently extended to volume correlative light-electron microscopy (vCLEM) [193] that may further expand PNN structural research opportunities. 

### 8.4. Technical Aspects of Introducing AI Tools in Biomedical Research

The rapid intercalation of AI in a range of methodologies raises the question of technical requirements both in terms of mathematical/IT competence and the available software/hardware equipment opportunities.

The development and tailoring of AI-based image analysis tools impose significant mathematical and coding competence requirements, restricting the scale of AI usage in current biomedical research. The bottleneck here is the development of user-friendly interfaces allowing biologists to access the AI instruments. To that end, ilastik [194] and Fiji Labkit [195] are “light solution” examples of machine learning-driven software for simplified biomedical image analysis that do not require large-scale ground-truth datasets, long learning times, and coding experience. Interestingly, Labkit is now also compatible with Imaris (Oxford Instruments)—another powerful image analysis software pack rapidly expanding towards AI implementation [196].

In contrast to “light tools” like ilastik and Labkit, the training step of “full scale” machine learning tools usually takes some hours or days, depending on the graphics processing unit (GPU) performance and the size of the training dataset. The requirements for computational power and data storage depend on the size of the training dataset and the complexity of the artificial neural network architecture. The artificial neural network training can be performed both on the central processing unit (CPU) and on the GPU of a computer, but GPUs are much better suited for that purpose, having thousands of cores and therefore allowing for fast parallel computations. In that regard, the size of the GPU video random-access memory (VRAM, typically above 4 Gb) and the number of CUDA Cores within GPU are the two essential parameters for machine learning applications.

## 9. AI Tools in Brain Pathology Studies

Machine learning tools for medical image classification and analysis have been advancing rapidly over the last few years [197]. Among other applications, the cell counting problem has been addressed by several approaches [198,199,200]. In particular, the random forest models were used with convolutional neural networks to achieve minimal counting error values as compared to other machine learning-assisted cell counting solutions [198]. Importantly, low counting error values were achieved for the small training datasets that are often a crucial issue for biomedical samples. Another AI-assisted method performs cell counting as a regression task of learning an inner distance metric [200]. The method was used to detect both cell centers and boundaries. Of interest, it was shown to work efficiently on one cell line after being trained on a different cell line.

Brain tissue microscopy image analysis has been facilitated with AI tools in a number of studies [201,202,203]. One obvious trend in that direction is the combination of multiphoton microscopy with deep learning on unstained brain tissue samples for diagnostic applications.

Wang and co-authors took advantage of two-photon microscopy to identify the infarct core, peri-infarct area, and a remote area in a rat cerebral ischemia–reperfusion model [201]. The authors developed a deep learning model based on a convolutional neural network to automatically detect the location of injured neurons on unstained thin sections and fresh tissue. Furthermore, they applied deep learning-assisted two-photon microscopy to evaluate the ischemic regions based on tissue edema, two-photon-excited fluorescence signal intensity, as well as neuronal injury.

Chen and co-authors used multiphoton microscopy on label- and processing-free surgical specimens of human brain tissue as a novel method for rapid intraoperative diagnostics of infiltrating glioma cancer [203]. The authors applied deep learning to achieve high accuracy in distinguishing gray from white matter and cancer from non-cancer. Thirty-five specimens from 18 patients were selected by a neuropathologist for training two residual convolutional neural network (ResNet) models—one for grey versus white matter discrimination based on 2389 fields of view, and the other one for cancerous versus non-cancerous tissue based on 3909 fields of view.

Another study by Cai and co-authors does not deal with a pathology model but the proposed methodology may be very useful for research on a range of brain diseases [202]. The authors demonstrate that the artificial neural network RetinaNet model is highly efficient in classifying neurons and glia in microscopy images from the brain sections of mosaic analysis with double markers (MADM) mice. Notably, the method exhibited difficulties in classifying glial clusters and the problem was resolved by combining two RetinaNet models, one trained for single cells and the other for glial clusters. To diversify the training data, the authors used both confocal microscopy and a slide scanner. Moreover, genetically different MADM mice were used to generate the training dataset with different cellular densities. 

## 10. AI Tools for PNN Studies 

Current advances in transgenic animals, MRI and advanced large-scale microscopy techniques bring the neuroscience research far ahead towards the brain connectome studies making it possible to address connectivity, functioning and plasticity of larger neuronal ensembles and synaptic network mechanisms of complex cognitive functions like memory and decision-making [204,205]. The bottleneck for taking advantage of the rapidly growing biomedical datasets is the data annotation step that is still handled manually or semi-automatically for the majority of applications [14,42], which makes data analysis expensive, time-consuming and dependent on skilled professionals for annotation [206]. The rapid introduction of machine learning tools for t brain tissue image analysis has the potential to significantly accelerate the research area of brain functional morphology, including the ECM and PNN research [34,36,204]. The AI-assisted study of drug-induced conditioning by Traver and co-authors [205] provides an example of that kind.

Ciampi and co-authors aimed at developing a cell-counting deep learning-assisted method able to obtain highly accurate results from a dataset with weak multi-rater labels [34]. The authors used three publicly available annotated eukaryotic cell datasets and one dataset of synthetic images simulating bacterial cells for training with three converging neuronal network-based methods, i.e., segmentation-based S-UNet, detection-based FRCNN and density-based approaches DCSRNet. To overcome the training quality limitations imposed by raters’ disagreement, they introduced a second rescoring stage that was trained on a small multi-rater subset and refined the previously computed predictions. The resulting cell-counting method was tested on a PNN fluorescent microscopy dataset collected with a 10× objective from 25 brain sections and containing 34,000 annotated PNNs. The AI-assisted PNN counting procedure exploiting the redundant information of multi-rater data enhanced the accuracy level of the AI-assisted PNN analysis significantly [34]. The authors used the mean error of manual vs. AI-assisted counts (mean absolute counting error, MAE) as the main readout to evaluate how accurately the AI-assisted tools mimic the manual analysis. The models tended to correctly identify and count the PNNs found by more raters. The authors reported high variability in the MAE values between samples and suggested that dimmer PNNs in certain brain regions could be more difficult to detect both by AI and human experts.

This methodology was then used by the same authors for the deep learning-assisted analysis of their “Comprehensive atlas of perineuronal net distribution and colocalization with parvalbumin in the adult mouse brain” [36]. Two deep convolutional neural networks were trained with a dataset comprising roughly 0.67 million manually annotated PNNs and 0.16 million PV cells. Among other findings, the authors demonstrated the difference between primary and secondary sensory cortex areas regarding the probability of a PNN coating on PV+ cells. Furthermore, the authors used the Allen institute mouse brain connectivity atlas to demonstrate a high correlation between the “PNN energy” (PNN density weighted by the fluorescence intensity) and thalamic input strength in cortical layers 2/3, 4, and 5, and this effect was most prominent in layer 4, suggesting a functional connection between the PNN expression and thalamic input on PV+ interneurons. The authors speculate that the brain-wide comparison of very large PNN and PV cellular datasets with the public resources presented in their study would be further enhanced by the advent of spatial transcriptomics.

## 11. AI-Assisted PNN Mesh Tracing

**Figure 5:**
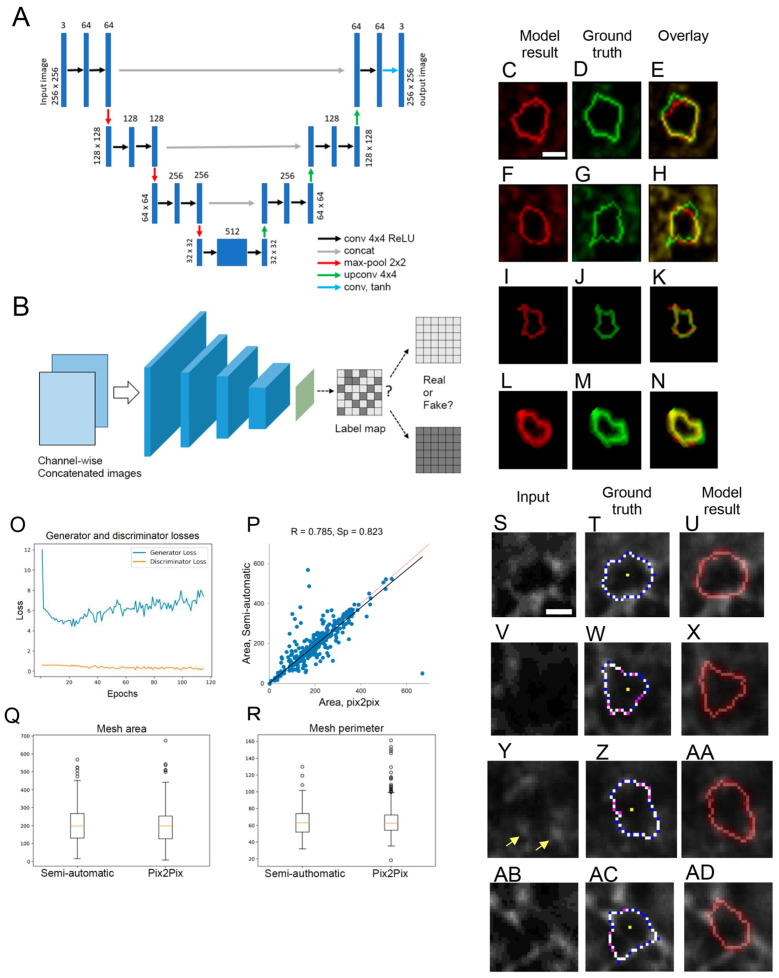
Machine learning model architecture and PNN mesh contour annotation results: (**A**) the U-Net architecture used as a generator; (**B**) the PatchGAN discriminator architecture; (**C**–**N**) comparison of the model result vs. ground truth for the same four meshes as in Figure 5; (**O**) the plot for losses during the model training; (**P**) the correlation plot for individual mesh area values, model results vs. ground truth. The values for Pearson’s and Spearman’s correlation coefficients are shown on top; (**Q**,**R**) the mean values for the mesh area (**Q**) and perimeter (**R**) for the model results and ground truth; (**S**–**AD**) examples of high contrast (**S**–**U**), blurred (**V**–**X**), polar (**Y**–**AA**) meshes and some meshes that were traced with the Model 1 more correctly then with the semi-automated method (**AB**–**AD**). The scale bar in (**C**,**S**) is 1 μm.

Here, we report on the development of a machine learning tool for tracing PNN single-mesh contours in high-resolution confocal images. We and others previously reported on a few methods for the annotation of PNN mesh geometry [14,39,42]. To further accelerate the high-content analysis of PNN high-resolution structural studies, we tried two different machine learning approaches—one using image-to-image translation with Pix2Pix (U-Net architecture used as a generator) [207] GAN [208] model (Model 1) (Figure 5A) and the other using image-to-contour translation with the same Pix2Pix model (Model 2) (for a detailed description please see the Supplementary Materials). The PatchGAN discriminator was used in both models, acting as a style/texture loss function, assuming independence between pixels separated from each other by more than a fragment diameter (Figure 5B) (see Supplementary Materials for further detail). The PFC PNN confocal dataset described in [42] was used with 7897 annotated PNN meshes (units). The dataset was divided randomly into an 80% training set and a 20% test set.

### Results and Discussion

**Figure 6:**
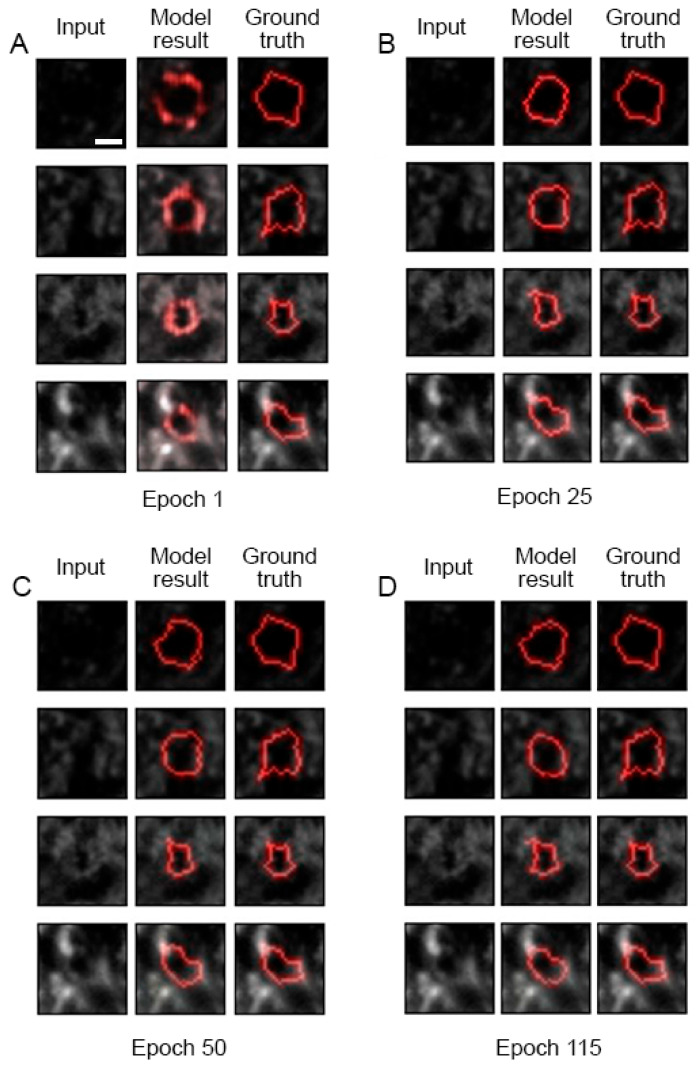
Model 1 output results during the learning process. Four meshes were randomly selected before learning and the results were snapshot at the epochs 1 (**A**), 25 (**B**), 50 (**C**), 115 (**D**). The scale bar in (**A**) is 0.5 μm.

The model learning progress for Model 1 is demonstrated in Figure 6 for epochs 1, 25, 50, 115 for four PNN meshes that were randomly selected before the model training and then tested at different epochs of the training process. An overlay of the ground-truth (semi-automated annotation) (Figure 5C,F,I,L) and model-derived (Figure 5D,G,J,M) contours demonstrates good matches for the two upper meshes (Figure 5E,H) and a nearly perfect match for the two lower meshes (Figure 5K,N).

The training process graph (Figure 5O) shows the Pix2Pix generator losses (blue) and the discriminator losses (yellow). The high values of the mesh area correlation coefficients between the model contours and ground truth (Figure 5P), together with a comparison of the area and perimeter mean values between the two annotation sets (Figure 5Q,R), support the conclusion that the mesh contour geometry generated by the machine learning tool is consistent with the training set values. The method generates the PNN mesh contours matching both the WFA fluorescence patterns and the ground-truth annotation not only for high-contrast meshes (Figure 5S–U) but also for the PNN meshes with blurred fluorescence patterns (Figure 5V–X). We previously described distinct patterns of the CSPG-WFA staining-intensity distribution along the mesh contour [14]. The Model 1 machine learning tool described here is able to recognize and properly trace polar meshes with vertex-enriched CSPG and weak or absent WFA staining fluorescence in between the vertices (Figure 5Y–AA). Interestingly, the Model 1 tool described here also provides examples of PNN meshes with erroneous tracing in the ground truth and much more accurate tracing produced by Model 1 (Figure 5AB–AD).

The alternative approach (Model 2) did not exhibit sufficient learning progress (Figure S11), leading to the conclusion that the image-to-contour translation is not applicable with this particular model architecture.

The main advantage of the proposed approach is its ability to process large amounts of data in a fully automatic mode that does not require human involvement. Pix2Pix is an end-to-end model, which means that it is trainable for solving complex tasks using raw data directly as an input without any manual feature extraction. Hence, the method can be used on a range of variable datasets.

In terms of downsides, the method requires appropriate expertise within the scientific team to adjust the model for solving a specific research task. To that end, complementing the tool with an easy-to-use interface would enable biologists to load additional images to fine-tune the model without having professional competence in machine learning.

The Model 1 with image-to-image translation Pix2Pix GAN architecture described here demonstrates the ability to automatically generate the PNN single-mesh contour annotation that may be valuable for the acceleration of high-resolution PNN image analysis for biomedical applications. In its present state, the model requires a local region of interest containing a PNN single mesh as an input image. The next step would be to further develop the tool to automatically detect and annotate the PNN mesh structure in whole neuronal cell confocal images.

## 12. Conclusions

The PNN microscopy field has been growing rapidly over the last few years and is demonstrating the value of imaging and image analysis techniques in several directions of brain pathology research. We are starting to gain insights into CNS disease mechanisms at the level of abnormalities in the delicate microstructure of synapses and the surrounding ECM. Quantitative image analysis plays a pivotal role in this. Live brain multiphoton imaging at the subcellular resolution provides hope that rePNN dynamics during synaptic network maturation, and in the adult brain, will be revealed in order to address the processes of memory, oxidative stress and the onset of disease states like schizophrenia and AD. The pharmacological targeting of PNN is attracting significant attention, and a range of microscopy techniques will definitely contribute towards further analyses.

The following trends may be important for the field development in near future:

- Implementation of high-throughput instrumental upgrades both in low and high-resolution microscopy to speed up the pipeline for the data collection;
- Transition from low-resolution microscopy meant for counting PNN numbers to high-resolution imaging aiming at insights into synaptic structure and function. Superresolution microscopy, multiphoton microscopy, correlative light–electron microscopy (CLEM) and electron tomography are instrumental for efficient progress along these lines;
- Expanding the range of quantitative image analysis methods in order to increase collected structural information and build high-resolution 3D models elucidating the structural basis of physiological functions and brain pathologies. Integrating the PNN data into connectomics research may be particularly fruitful;
- Implementation of AI instruments aimed at high-content unbiased quantitative microscopy data analysis and achieving new unprecedented levels of insight into PNN structure and function.

The dissection of causal connections between genes, biological macromolecules, and physiological functions has made a tremendous impact on the development of biomedicine based on molecular biology, biochemistry, behavioral and electrophysiology techniques, revealing a range of brain pathology mechanisms. Thus far, microscopy has often served to provide illustrations in support of mechanistic findings. The trend towards quantitative microscopy aimed at novel mechanistic insights is gaining impetus as a result of the exploding AI research field. We currently face demands from both society and the scientific community for an understanding of the scale and direction of future changes resulting from the ongoing AI technological revolution. We hope that the present study could contribute towards that purpose, from a brain pathology-related PNN microscopy perspective.

## Supplementary Materials

The following supporting information can be downloaded at: https://www.mdpi.com/article/10.3390/ijms25084227/s1 [209,210,211,212,213,214].

## References

[1] Fawcett J.W., Oohashi T., Pizzorusso T. (2019). The roles of perineuronal nets and the perinodal extracellular matrix in neuronal function. Nat. Rev. Neurosci..

[2] Dong Y., Zhao K., Qin X., Du G., Gao L. (2023). The mechanisms of perineuronal net abnormalities in contributing aging and neurological diseases. Ageing Res. Rev..

[3] Pantazopoulos H., Katsel P., Haroutunian V., Chelini G., Klengel T., Berretta S. (2021). Molecular signature of extracellular matrix pathology in schizophrenia. Eur. J. Neurosci..

[4] Carceller H., Gramuntell Y., Klimczak P., Nacher J. (2023). Perineuronal Nets: Subtle Structures with Large Implications. Neuroscientist.

[5] Zeug A., Stawarski M., Bieganska K., Korotchenko S., Wlodarczyk J., Dityatev A., Ponimaskin E. (2014). Current microscopic methods for the neural ECM analysis. Progress in Brain Research.

[6] Celio M.R., Spreafico R., De Biasi S., Vitellaro-Zuccarello L. (1998). Perineuronal nets: Past and present. Trends Neurosci..

[7] Ruoslahti E. (1996). Brain extracellular matrix. Glycobiology.

[8] Yamaguchi Y. (2000). Lecticans: Organizers of the brain extracellular matrix. Cell. Mol. Life Sci..

[9] Deepa S.S., Carulli D., Galtrey C., Rhodes K., Fukuda J., Mikami T., Sugahara K., Fawcett J.W. (2006). Composition of Perineuronal Net Extracellular Matrix in Rat Brain. J. Biol. Chem..

[10] Kwok J.C.F., Dick G., Wang D., Fawcett J.W. (2011). Extracellular matrix and perineuronal nets in CNS repair. Dev. Neurobiol..

[11] Eill G.J., Sinha A., Morawski M., Viapiano M.S., Matthews R.T. (2020). The protein tyrosine phosphatase RPTPζ/phosphacan is critical for perineuronal net structure. J. Biol. Chem..

[12] Carulli D., De Winter F., Verhaagen J. (2021). Semaphorins in Adult Nervous System Plasticity and Disease. Front. Synaptic Neurosci..

[13] Ferrer-Ferrer M., Dityatev A. (2018). Shaping Synapses by the Neural Extracellular Matrix. Front. Neuroanat..

[14] Arnst N., Kuznetsova S., Lipachev N., Shaikhutdinov N., Melnikova A., Mavlikeev M., Uvarov P., Baltina T.V., Rauvala H., Osin Y.N. (2016). Spatial patterns and cell surface clusters in perineuronal nets. Brain Res..

[15] Bosiacki M., Gąssowska-Dobrowolska M., Kojder K., Fabiańska M., Jeżewski D., Gutowska I., Lubkowska A. (2019). Perineuronal Nets and Their Role in Synaptic Homeostasis. Int. J. Mol. Sci..

[16] Lander C., Zhang H., Hockfield S. (1998). Neurons Produce a Neuronal Cell Surface-Associated Chondroitin Sulfate Proteoglycan. J. Neurosci..

[17] Brückner G., Kacza J., Grosche J. (2003). Perineuronal Nets Characterized by Vital Labelling, Confocal and Electron Microscopy in Organotypic Slice Cultures of Rat Parietal Cortex and Hippocampus. J. Mol. Histol..

[18] McRae P.A., Rocco M.M., Kelly G., Brumberg J.C., Matthews R.T. (2007). Sensory Deprivation Alters Aggrecan and Perineuronal Net Expression in the Mouse Barrel Cortex. J. Neurosci..

[19] Carulli D., Pizzorusso T., Kwok J.C.F., Putignano E., Poli A., Forostyak S., Andrews M.R., Deepa S.S., Glant T.T., Fawcett J.W. (2010). Animals lacking link protein have attenuated perineuronal nets and persistent plasticity. Brain.

[20] Lipachev N., Melnikova A., Fedosimova S., Arnst N., Kochneva A., Shaikhutdinov N., Dvoeglazova A., Titova A., Mavlikeev M., Aganov A. (2022). Postnatal development of the microstructure of cortical GABAergic synapses and perineuronal nets requires sensory input. Neurosci. Res..

[21] Pizzorusso T., Medini P., Berardi N., Chierzi S., Fawcett J.W., Maffei L. (2002). Reactivation of Ocular Dominance Plasticity in the Adult Visual Cortex. Science.

[22] Miyata S., Kitagawa H. (2015). Mechanisms for modulation of neural plasticity and axon regeneration by chondroitin sulphate. J. Biochem..

[23] Hou X., Yoshioka N., Tsukano H., Sakai A., Miyata S., Watanabe Y., Yanagawa Y., Sakimura K., Takeuchi K., Kitagawa H. (2017). Chondroitin Sulfate Is Required for Onset and Offset of Critical Period Plasticity in Visual Cortex. Sci. Rep..

[24] Dityatev A., Rusakov D.A. (2011). Molecular signals of plasticity at the tetrapartite synapse. Curr. Opin. Neurobiol..

[25] Carulli D., Broersen R., De Winter F., Muir E.M., Mešković M., De Waal M., De Vries S., Boele H.-J., Canto C.B., De Zeeuw C.I. (2020). Cerebellar plasticity and associative memories are controlled by perineuronal nets. Proc. Natl. Acad. Sci. USA.

[26] Frischknecht R., Heine M., Perrais D., Seidenbecher C.I., Choquet D., Gundelfinger E.D. (2009). Brain extracellular matrix affects AMPA receptor lateral mobility and short-term synaptic plasticity. Nat. Neurosci..

[27] Tewari B.P., Chaunsali L., Campbell S.L., Patel D.C., Goode A.E., Sontheimer H. (2018). Perineuronal nets decrease membrane capacitance of peritumoral fast spiking interneurons in a model of epilepsy. Nat. Commun..

[28] Tewari B., Woo A., Prim C., Chaunsali L., Kimbrough I., Engel K., Browning J., Campbell S., Sontheimer H. (2023). Perineuronal Nets Support Astrocytic Ion and Glutamate Homeostasis at Tripartite Synapses. Res. Sq..

[29] Dityatev A., Wehrle-Haller B., Pitkänen A. (2014). Preface. Progress in Brain Research.

[30] Fawcett J.W., Fyhn M., Jendelova P., Kwok J.C.F., Ruzicka J., Sorg B.A. (2022). The extracellular matrix and perineuronal nets in memory. Mol. Psychiatry.

[31] Mueller-Buehl C., Wegrzyn D., Bauch J., Faissner A. (2023). Regulation of the E/I-balance by the neural matrisome. Front. Mol. Neurosci..

[32] Slaker M., Barnes J., Sorg B.A., Grimm J.W. (2016). Impact of Environmental Enrichment on Perineuronal Nets in the Prefrontal Cortex following Early and Late Abstinence from Sucrose Self-Administration in Rats. PLoS ONE.

[33] Lipachev N., Arnst N., Melnikova A., Jäälinoja H., Kochneva A., Zhigalov A., Kulesskaya N., Aganov A.V., Mavlikeev M., Rauvala H. (2019). Quantitative changes in perineuronal nets in development and posttraumatic condition. J. Mol. Histol..

[34] Ciampi L., Carrara F., Totaro V., Mazziotti R., Lupori L., Santiago C., Amato G., Pizzorusso T., Gennaro C. (2022). Learning to count biological structures with raters’ uncertainty. Med. Image Anal..

[35] Slaker M.L., Harkness J.H., Sorg B.A. (2016). A standardized and automated method of perineuronal net analysis using Wisteria floribunda agglutinin staining intensity. IBRO Rep..

[36] Lupori L., Totaro V., Cornuti S., Ciampi L., Carrara F., Grilli E., Viglione A., Tozzi F., Putignano E., Mazziotti R. (2023). A comprehensive atlas of perineuronal net distribution and colocalization with parvalbumin in the adult mouse brain. Cell Rep..

[37] Tippani M., Divecha H.R., Catallini J.L., Kwon S.H., Weber L.M., Spangler A., Jaffe A.E., Hyde T.M., Kleinman J.E., Hicks S.C. (2023). *VistoSeg*: Processing utilities for high-resolution images for spatially resolved transcriptomics data. Biol. Imaging.

[38] Brückner G., Szeöke S., Pavlica S., Grosche J., Kacza J. (2006). Axon initial segment ensheathed by extracellular matrix in perineuronal nets. Neuroscience.

[39] Dzyubenko E., Manrique-Castano D., Kleinschnitz C., Faissner A., Hermann D.M. (2018). Topological remodeling of cortical perineuronal nets in focal cerebral ischemia and mild hypoperfusion. Matrix Biol..

[40] Dzyubenko E., Willig K.I., Yin D., Sardari M., Tokmak E., Labus P., Schmermund B., Hermann D.M. (2023). Structural changes in perineuronal nets and their perforating GABAergic synapses precede motor coordination recovery post stroke. J. Biomed. Sci..

[41] Sigal Y.M., Bae H., Bogart L.J., Hensch T.K., Zhuang X. (2019). Structural maturation of cortical perineuronal nets and their perforating synapses revealed by superresolution imaging. Proc. Natl. Acad. Sci. USA.

[42] Kaushik R., Lipachev N., Matuszko G., Kochneva A., Dvoeglazova A., Becker A., Paveliev M., Dityatev A. (2021). Fine structure analysis of perineuronal nets in the ketamine model of schizophrenia. Eur. J. Neurosci..

[43] Lev-Ram V., Lemieux S.P., Deerinck T.J., Bushong E.A., Toyama B.H., Perez A., Pritchard D.R., Park S.K.R., McClatchy D.B., Savas J.N. (2023). Do perineuronal nets stabilize the engram of a Synaptic Circuit?. Neuroscience.

[44] Tansley S., Gu N., Guzmán A.U., Cai W., Wong C., Lister K.C., Muñoz-Pino E., Yousefpour N., Roome R.B., Heal J. (2022). Microglia-mediated degradation of perineuronal nets promotes pain. Science.

[45] Vo T., Carulli D., Ehlert E.M.E., Kwok J.C.F., Dick G., Mecollari V., Moloney E.B., Neufeld G., De Winter F., Fawcett J.W. (2013). The chemorepulsive axon guidance protein semaphorin3A is a constituent of perineuronal nets in the adult rodent brain. Mol. Cell. Neurosci..

[46] Carulli D., Rhodes K.E., Fawcett J.W. (2007). Upregulation of aggrecan, link protein 1, and hyaluronan synthases during formation of perineuronal nets in the rat cerebellum. J. Comp. Neurol..

[47] Galtrey C.M., Kwok J.C.F., Carulli D., Rhodes K.E., Fawcett J.W. (2008). Distribution and synthesis of extracellular matrix proteoglycans, hyaluronan, link proteins and tenascin-R in the rat spinal cord. Eur. J. Neurosci..

[48] Pantazopoulos H., Lange N., Hassinger L., Berretta S. (2006). Subpopulations of neurons expressing parvalbumin in the human amygdala. J. Comp. Neurol..

[49] Pantazopoulos H., Murray E.A., Berretta S. (2008). Total number, distribution, and phenotype of cells expressing chondroitin sulfate proteoglycans in the normal human amygdala. Brain Res..

[50] Weinrich L., Sonntag M., Arendt T., Morawski M. (2018). Neuroanatomical characterization of perineuronal net components in the human cochlear nucleus and superior olivary complex. Hear. Res..

[51] Jäger C., Lendvai D., Seeger G., Brückner G., Matthews R.T., Arendt T., Alpár A., Morawski M. (2013). Perineuronal and perisynaptic extracellular matrix in the human spinal cord. Neuroscience.

[52] Rahmani R., Rambarack N., Singh J., Constanti A., Ali A.B. (2023). Age-Dependent Sex Differences in Perineuronal Nets in an APP Mouse Model of Alzheimer’s Disease Are Brain Region-Specific. Int. J. Mol. Sci..

[53] Pantazopoulos H., Woo T.-U.W., Lim M.P., Lange N., Berretta S. (2010). Extracellular Matrix-Glial Abnormalities in the Amygdala and Entorhinal Cortex of Subjects Diagnosed with Schizophrenia. Arch. Gen. Psychiatry.

[54] Pantazopoulos H., Markota M., Jaquet F., Ghosh D., Wallin A., Santos A., Caterson B., Berretta S. (2015). Aggrecan and chondroitin-6-sulfate abnormalities in schizophrenia and bipolar disorder: A postmortem study on the amygdala. Transl. Psychiatry.

[55] Kilonzo V.W., Sweet R.A., Glausier J.R., Pitts M.W. (2020). Deficits in Glutamic Acid Decarboxylase 67 Immunoreactivity, Parvalbumin Interneurons, and Perineuronal Nets in the Inferior Colliculus of Subjects with Schizophrenia. Schizophr. Bull..

[56] Mauney S.A., Athanas K.M., Pantazopoulos H., Shaskan N., Passeri E., Berretta S., Woo T.-U.W. (2013). Developmental Pattern of Perineuronal Nets in the Human Prefrontal Cortex and Their Deficit in Schizophrenia. Biol. Psychiatry.

[57] Romberg C., Yang S., Melani R., Andrews M.R., Horner A.E., Spillantini M.G., Bussey T.J., Fawcett J.W., Pizzorusso T., Saksida L.M. (2013). Depletion of Perineuronal Nets Enhances Recognition Memory and Long-Term Depression in the Perirhinal Cortex. J. Neurosci..

[58] Schmidt S., Stapf C., Schmutzler S., Lachmann I., Arendt T., Holzer M., Sonntag M., Morawski M. (2021). Aggrecan modulates the expression and phosphorylation of tau in a novel bigenic TauP301L—*Acan* mouse model. Eur. J. Neurosci..

[59] Yang S., Cacquevel M., Saksida L.M., Bussey T.J., Schneider B.L., Aebischer P., Melani R., Pizzorusso T., Fawcett J.W., Spillantini M.G. (2015). Perineuronal net digestion with chondroitinase restores memory in mice with tau pathology. Exp. Neurol..

[60] Yang S., Hilton S., Alves J.N., Saksida L.M., Bussey T., Matthews R.T., Kitagawa H., Spillantini M.G., Kwok J.C.F., Fawcett J.W. (2017). Antibody recognizing 4-sulfated chondroitin sulfate proteoglycans restores memory in tauopathy-induced neurodegeneration. Neurobiol. Aging.

[61] Pantazopoulos H., Gisabella B., Rexrode L., Benefield D., Yildiz E., Seltzer P., Valeri J., Chelini G., Reich A., Ardelt M. (2020). Circadian Rhythms of Perineuronal Net Composition. eNeuro.

[62] Valeri J., Stiplosek C., O’Donovan S.M., Sinclair D., Grant K., Bollavarapu R., Platt D.M., Stockmeier C.A., Gisabella B., Pantazopoulos H. (2023). Extracellular Matrix Abnormalities in the Hippocampus of Subjects with Substance Use Disorder. Addict. Med..

[63] Gáti G., Morawski M., Lendvai D., Matthews R.T., Jäger C., Zachar G., Arendt T., Alpár A. (2010). Chondroitin sulphate proteoglycan-based perineuronal net establishment is largely activity-independent in chick visual system. J. Chem. Neuroanat..

[64] Rosenzweig E.S., Salegio E.A., Liang J.J., Weber J.L., Weinholtz C.A., Brock J.H., Moseanko R., Hawbecker S., Pender R., Cruzen C.L. (2019). Chondroitinase improves anatomical and functional outcomes after primate spinal cord injury. Nat. Neurosci..

[65] Carulli D., Rhodes K.E., Brown D.J., Bonnert T.P., Pollack S.J., Oliver K., Strata P., Fawcett J.W. (2006). Composition of perineuronal nets in the adult rat cerebellum and the cellular origin of their components. J. Comp. Neurol..

[66] Kwok J.C.F., Carulli D., Fawcett J.W. (2010). *In vitro* modeling of perineuronal nets: Hyaluronan synthase and link protein are necessary for their formation and integrity. J. Neurochem..

[67] Dick G., Tan C.L., Alves J.N., Ehlert E.M.E., Miller G.M., Hsieh-Wilson L.C., Sugahara K., Oosterhof A., Van Kuppevelt T.H., Verhaagen J. (2013). Semaphorin 3A Binds to the Perineuronal Nets via Chondroitin Sulfate Type E Motifs in Rodent Brains. J. Biol. Chem..

[68] Wang D., Ichiyama R.M., Zhao R., Andrews M.R., Fawcett J.W. (2011). Chondroitinase Combined with Rehabilitation Promotes Recovery of Forelimb Function in Rats with Chronic Spinal Cord Injury. J. Neurosci..

[69] Štepánková K., Chudíčková M., Šimková Z., Martinez-Varea N., Kubinová Š., Urdzíková L.M., Jendelová P., Kwok J.C.F. (2023). Low oral dose of 4-methylumbelliferone reduces glial scar but is insufficient to induce functional recovery after spinal cord injury. Sci. Rep..

[70] Lépine M., Douceau S., Devienne G., Prunotto P., Lenoir S., Regnauld C., Pouettre E., Piquet J., Lebouvier L., Hommet Y. (2022). Parvalbumin interneuron-derived tissue-type plasminogen activator shapes perineuronal net structure. BMC Biol..

[71] Perna J., Lu J., Mullen B., Liu T., Tjia M., Weiser S., Ackman J., Zuo Y. (2021). Perinatal Penicillin Exposure Affects Cortical Development and Sensory Processing. Front. Mol. Neurosci..

[72] Roura-Martínez D., Díaz-Bejarano P., Ucha M., Paiva R.R., Ambrosio E., Higuera-Matas A. (2020). Comparative analysis of the modulation of perineuronal nets in the prefrontal cortex of rats during protracted withdrawal from cocaine, heroin and sucrose self-administration. Neuropharmacology.

[73] Xue Y.-X., Xue L.-F., Liu J.-F., He J., Deng J.-H., Sun S.-C., Han H.-B., Luo Y.-X., Xu L.-Z., Wu P. (2014). Depletion of Perineuronal Nets in the Amygdala to Enhance the Erasure of Drug Memories. J. Neurosci..

[74] Marchand A., Schwartz C. (2020). Perineuronal net expression in the brain of a hibernating mammal. Brain Struct. Funct..

[75] Ueno H., Suemitsu S., Murakami S., Kitamura N., Wani K., Takahashi Y., Matsumoto Y., Okamoto M., Ishihara T. (2019). Alteration of Extracellular Matrix Molecules and Perineuronal Nets in the Hippocampus of Pentylenetetrazol-Kindled Mice. Neural Plast..

[76] Enwright J.F., Sanapala S., Foglio A., Berry R., Fish K.N., Lewis D.A. (2016). Reduced Labeling of Parvalbumin Neurons and Perineuronal Nets in the Dorsolateral Prefrontal Cortex of Subjects with Schizophrenia. Neuropsychopharmacology.

[77] Steullet P., Cabungcal J.-H., Bukhari S.A., Ardelt M.I., Pantazopoulos H., Hamati F., Salt T.E., Cuenod M., Do K.Q., Berretta S. (2018). The thalamic reticular nucleus in schizophrenia and bipolar disorder: Role of parvalbumin-expressing neuron networks and oxidative stress. Mol. Psychiatry.

[78] Rey C.C., Robert V., Bouisset G., Loisy M., Lopez S., Cattaud V., Lejards C., Piskorowski R.A., Rampon C., Chevaleyre V. (2022). Altered inhibitory function in hippocampal CA2 contributes in social memory deficits in Alzheimer’s mouse model. iScience.

[79] Boggio E.M., Ehlert E.M., Lupori L., Moloney E.B., De Winter F., Vander Kooi C.W., Baroncelli L., Mecollari V., Blits B., Fawcett J.W. (2019). Inhibition of Semaphorin3A Promotes Ocular Dominance Plasticity in the Adult Rat Visual Cortex. Mol. Neurobiol..

[80] Dubisova J., Burianova J.S., Svobodova L., Makovicky P., Martinez-Varea N., Cimpean A., Fawcett J.W., Kwok J.C.F., Kubinova S. (2022). Oral treatment of 4-methylumbelliferone reduced perineuronal nets and improved recognition memory in mice. Brain Res. Bull..

[81] Blosa M., Bursch C., Weigel S., Holzer M., Jäger C., Janke C., Matthews R.T., Arendt T., Morawski M. (2016). Reorganization of Synaptic Connections and Perineuronal Nets in the Deep Cerebellar Nuclei of *Purkinje Cell Degeneration* Mutant Mice. Neural Plast..

[82] Shah A., Lodge D.J. (2013). A loss of hippocampal perineuronal nets produces deficits in dopamine system function: Relevance to the positive symptoms of schizophrenia. Transl. Psychiatry.

[83] Napoli D., Lupori L., Mazziotti R., Sagona G., Bagnoli S., Samad M., Sacramento E.K., Kirkpartick J., Putignano E., Chen S. (2020). MiR-29 coordinates age-dependent plasticity brakes in the adult visual cortex. EMBO Rep..

[84] Lensjø K.K., Lepperød M.E., Dick G., Hafting T., Fyhn M. (2017). Removal of Perineuronal Nets Unlocks Juvenile Plasticity Through Network Mechanisms of Decreased Inhibition and Increased Gamma Activity. J. Neurosci..

[85] Erchova I., Vasalauskaite A., Longo V., Sengpiel F. (2017). Enhancement of visual cortex plasticity by dark exposure. Philos. Trans. R. Soc. B Biol. Sci..

[86] Irvine S., Kwok J. (2018). Perineuronal Nets in Spinal Motoneurones: Chondroitin Sulphate Proteoglycan around Alpha Motoneurones. Int. J. Mol. Sci..

[87] Dauth S., Grevesse T., Pantazopoulos H., Campbell P.H., Maoz B.M., Berretta S., Parker K.K. (2016). Extracellular matrix protein expression is brain region dependent. J. Comp. Neurol..

[88] Baidoe-Ansah D., Sakib S., Jia S., Mirzapourdelavar H., Strackeljan L., Fischer A., Aleshin S., Kaushik R., Dityatev A. (2022). Aging-Associated Changes in Cognition, Expression and Epigenetic Regulation of Chondroitin 6-Sulfotransferase Chst3. Cells.

[89] Benbenishty A., Peled-Hajaj S., Krishnaswamy V.R., Har-Gil H., Havusha-Laufer S., Ruggiero A., Slutsky I., Blinder P., Sagi I. (2023). Longitudinal in vivo imaging of perineuronal nets. Neurophotonics.

[90] Lemieux S.P., Lev-Ram V., Tsien R.Y., Ellisman M.H. (2023). Perineuronal nets and the neuronal extracellular matrix can be imaged by genetically encoded labeling of HAPLN1 in vitro and in vivo. Neuroscience.

[91] Carstens K.E., Phillips M.L., Pozzo-Miller L., Weinberg R.J., Dudek S.M. (2016). Perineuronal Nets Suppress Plasticity of Excitatory Synapses on CA2 Pyramidal Neurons. J. Neurosci..

[92] O’Connor A.M., Burton T.J., Mansuri H., Hand G.R., Leamey C.A., Sawatari A. (2019). Environmental Enrichment From Birth Impacts Parvalbumin Expressing Cells and Wisteria Floribunda Agglutinin Labelled Peri-Neuronal Nets within the Developing Murine Striatum. Front. Neuroanat..

[93] Carulli D., Foscarin S., Faralli A., Pajaj E., Rossi F. (2013). Modulation of semaphorin3A in perineuronal nets during structural plasticity in the adult cerebellum. Mol. Cell. Neurosci..

[94] Wegrzyn D., Freund N., Faissner A., Juckel G. (2021). Poly I:C Activated Microglia Disrupt Perineuronal Nets and Modulate Synaptic Balance in Primary Hippocampal Neurons in vitro. Front. Synaptic Neurosci..

[95] Van’t Spijker H.M., Rowlands D., Rossier J., Haenzi B., Fawcett J.W., Kwok J.C.F. (2019). Neuronal Pentraxin 2 Binds PNNs and Enhances PNN Formation. Neural Plast..

[96] Crapser J.D., Ochaba J., Soni N., Reidling J.C., Thompson L.M., Green K.N. (2020). Microglial depletion prevents extracellular matrix changes and striatal volume reduction in a model of Huntington’s disease. Brain.

[97] Stoyanov S., Sun W., Düsedau H.P., Cangalaya C., Choi I., Mirzapourdelavar H., Baidoe-Ansah D., Kaushik R., Neumann J., Dunay I.R. (2021). Attenuation of the extracellular matrix restores microglial activity during the early stage of amyloidosis. Glia.

[98] Lendvai D., Morawski M., Négyessy L., Gáti G., Jäger C., Baksa G., Glasz T., Attems J., Tanila H., Arendt T. (2013). Neurochemical mapping of the human hippocampus reveals perisynaptic matrix around functional synapses in Alzheimer’s disease. Acta Neuropathol..

[99] Alcaide J., Guirado R., Crespo C., Blasco-Ibáñez J.M., Varea E., Sanjuan J., Nacher J. (2019). Alterations of perineuronal nets in the dorsolateral prefrontal cortex of neuropsychiatric patients. Int. J. Bipolar Disord..

[100] Matuszko G., Curreli S., Kaushik R., Becker A., Dityatev A. (2017). Extracellular matrix alterations in the ketamine model of schizophrenia. Neuroscience.

[101] Allgäuer L., Cabungcal J.-H., Yzydorczyk C., Do K.Q., Dwir D. (2023). Low protein-induced intrauterine growth restriction as a risk factor for schizophrenia phenotype in a rat model: Assessing the role of oxidative stress and neuroinflammation interaction. Transl. Psychiatry.

[102] Slaker M.L., Jorgensen E.T., Hegarty D.M., Liu X., Kong Y., Zhang F., Linhardt R.J., Brown T.E., Aicher S.A., Sorg B.A. (2018). Cocaine Exposure Modulates Perineuronal Nets and Synaptic Excitability of Fast-Spiking Interneurons in the Medial Prefrontal Cortex. eNeuro.

[103] Wingert J.C., Anguiano J.N., Ramos J.D., Blacktop J.M., Gonzalez A.E., Churchill L., Sorg B.A. (2023). Enhanced expression of parvalbumin and perineuronal nets in the medial prefrontal cortex after extended-access cocaine self-administration in rats. Addict. Biol..

[104] Sanchez-Hernandez A., Nicolas C., Gil-Miravet I., Guarque-Chabrera J., Solinas M., Miquel M. (2021). Time-dependent regulation of perineuronal nets in the cerebellar cortex during abstinence of cocaine-self administration. Psychopharmacology.

[105] Jorgensen E.T., Gonzalez A.E., Harkness J.H., Hegarty D.M., Thakar A., Burchi D.J., Aadland J.A., Aicher S.A., Sorg B.A., Brown T.E. (2021). Cocaine memory reactivation induces functional adaptations within parvalbumin interneurons in the rat medial prefrontal cortex. Addict. Biol..

[106] Carbo-Gas M., Moreno-Rius J., Guarque-Chabrera J., Vazquez-Sanroman D., Gil-Miravet I., Carulli D., Hoebeek F., De Zeeuw C., Sanchis-Segura C., Miquel M. (2017). Cerebellar perineuronal nets in cocaine-induced pavlovian memory: Site matters. Neuropharmacology.

[107] Vazquez-Sanroman D., Leto K., Cerezo-Garcia M., Carbo-Gas M., Sanchis-Segura C., Carulli D., Rossi F., Miquel M. (2015). The cerebellum on cocaine: Plasticity and metaplasticity. Addict. Biol..

[108] Forostyak S., Forostyak O., Kwok J.C.F., Romanyuk N., Rehorova M., Kriska J., Dayanithi G., Raha-Chowdhury R., Jendelova P., Anderova M. (2020). Transplantation of Neural Precursors Derived from Induced Pluripotent Cells Preserve Perineuronal Nets and Stimulate Neural Plasticity in ALS Rats. Int. J. Mol. Sci..

[109] Yang T., Hu S., Chang W.-C., Kao H.-Y., Wang Y. (2022). Perineuronal Nets Degradation and Parvalbumin Interneuron Loss in a Mouse Model of DEPDC5-Related Epilepsy. Dev. Neurosci..

[110] Giamanco K.A., Morawski M., Matthews R.T. (2010). Perineuronal net formation and structure in aggrecan knockout mice. Neuroscience.

[111] Bertocchi I., Mele P., Ferrero G., Oberto A., Carulli D., Eva C. (2021). NPY-Y1 receptor signaling controls spatial learning and perineuronal net expression. Neuropharmacology.

[112] Rowlands D., Lensjø K.K., Dinh T., Yang S., Andrews M.R., Hafting T., Fyhn M., Fawcett J.W., Dick G. (2018). Aggrecan Directs Extracellular Matrix-Mediated Neuronal Plasticity. J. Neurosci..

[113] Ruzicka J., Dalecka M., Safrankova K., Peretti D., Jendelova P., Kwok J.C.F., Fawcett J.W. (2022). Perineuronal nets affect memory and learning after synapse withdrawal. Transl. Psychiatry.

[114] Sonntag M., Blosa M., Schmidt S., Reimann K., Blum K., Eckrich T., Seeger G., Hecker D., Schick B., Arendt T. (2018). Synaptic coupling of inner ear sensory cells is controlled by brevican-based extracellular matrix baskets resembling perineuronal nets. BMC Biol..

[115] Cannarozzo C., Rubiolo A., Casarotto P., Castrén E. (2023). Ketamine and its metabolite 2*R*,6*R*-hydroxynorketamine promote ocular dominance plasticity and release tropomyosin-related kinase B from inhibitory control without reducing perineuronal nets enwrapping parvalbumin interneurons. Eur. J. Neurosci..

[116] Gogolla N., Caroni P., Lüthi A., Herry C. (2009). Perineuronal Nets Protect Fear Memories from Erasure. Science.

[117] Shi W., Wei X., Wang X., Du S., Liu W., Song J., Wang Y. (2019). Perineuronal nets protect long-term memory by limiting activity-dependent inhibition from parvalbumin interneurons. Proc. Natl. Acad. Sci. USA.

[118] Poli A., Viglione A., Mazziotti R., Totaro V., Morea S., Melani R., Silingardi D., Putignano E., Berardi N., Pizzorusso T. (2023). Selective Disruption of Perineuronal Nets in Mice Lacking Crtl1 is Sufficient to Make Fear Memories Susceptible to Erasure. Mol. Neurobiol..

[119] Cabungcal J.-H., Steullet P., Morishita H., Kraftsik R., Cuenod M., Hensch T.K., Do K.Q. (2013). Perineuronal nets protect fast-spiking interneurons against oxidative stress. Proc. Natl. Acad. Sci. USA.

[120] Morawski M., Brückner M.K., Riederer P., Brückner G., Arendt T. (2004). Perineuronal nets potentially protect against oxidative stress. Exp. Neurol..

[121] Guirado R., Perez-Rando M., Sanchez-Matarredona D., Castrén E., Nacher J. (2014). Chronic fluoxetine treatment alters the structure, connectivity and plasticity of cortical interneurons. Int. J. Neuropsychopharmacol..

[122] Umemori J., Winkel F., Castrén E., Karpova N.N. (2015). Distinct effects of perinatal exposure to fluoxetine or methylmercury on parvalbumin and perineuronal nets, the markers of critical periods in brain development. Int. J. Dev. Neurosci..

[123] Jetsonen E., Didio G., Winkel F., Llach Pou M., Boj C., Kuczynski-Noyau L., Võikar V., Guirado R., Taira T., Lauri S.E. (2023). Activation of TrkB in Parvalbumin interneurons is required for the promotion of reversal learning in spatial and fear memory by antidepressants. Neuropsychopharmacology.

[124] Murthy S., Kane G.A., Katchur N.J., Lara Mejia P.S., Obiofuma G., Buschman T.J., McEwen B.S., Gould E. (2019). Perineuronal Nets, Inhibitory Interneurons, and Anxiety-Related Ventral Hippocampal Neuronal Oscillations Are Altered by Early Life Adversity. Biol. Psychiatry.

[125] Dityatev A., Brückner G., Dityateva G., Grosche J., Kleene R., Schachner M. (2007). Activity-dependent formation and functions of chondroitin sulfate-rich extracellular matrix of perineuronal nets. Dev. Neurobiol..

[126] Strackeljan L., Baczynska E., Cangalaya C., Baidoe-Ansah D., Wlodarczyk J., Kaushik R., Dityatev A. (2021). Microglia Depletion-Induced Remodeling of Extracellular Matrix and Excitatory Synapses in the Hippocampus of Adult Mice. Cells.

[127] Foscarin S., Ponchione D., Pajaj E., Leto K., Gawlak M., Wilczynski G.M., Rossi F., Carulli D. (2011). Experience-Dependent Plasticity and Modulation of Growth Regulatory Molecules at Central Synapses. PLoS ONE.

[128] Yang S., Gigout S., Molinaro A., Naito-Matsui Y., Hilton S., Foscarin S., Nieuwenhuis B., Tan C.L., Verhaagen J., Pizzorusso T. (2021). Chondroitin 6-sulphate is required for neuroplasticity and memory in ageing. Mol. Psychiatry.

[129] Wegrzyn D., Manitz M., Kostka M., Freund N., Juckel G., Faissner A. (2021). Poly I:C-induced maternal immune challenge reduces perineuronal net area and raises spontaneous network activity of hippocampal neurons in vitro. Eur. J. Neurosci..

[130] Gottschling C., Wegrzyn D., Denecke B., Faissner A. (2019). Elimination of the four extracellular matrix molecules tenascin-C, tenascin-R, brevican and neurocan alters the ratio of excitatory and inhibitory synapses. Sci. Rep..

[131] Morawski M., Dityatev A., Hartlage-Rübsamen M., Blosa M., Holzer M., Flach K., Pavlica S., Dityateva G., Grosche J., Brückner G. (2014). Tenascin-R promotes assembly of the extracellular matrix of perineuronal nets via clustering of aggrecan. Philos. Trans. R. Soc. B Biol. Sci..

[132] Klimczak P., Rizzo A., Castillo-Gómez E., Perez-Rando M., Gramuntell Y., Beltran M., Nacher J. (2021). Parvalbumin Interneurons and Perineuronal Nets in the Hippocampus and Retrosplenial Cortex of Adult Male Mice After Early Social Isolation Stress and Perinatal NMDA Receptor Antagonist Treatment. Front. Synaptic Neurosci..

[133] Cope E.C., Zych A.D., Katchur N.J., Waters R.C., Laham B.J., Diethorn E.J., Park C.Y., Meara W.R., Gould E. (2022). Atypical perineuronal nets in the CA2 region interfere with social memory in a mouse model of social dysfunction. Mol. Psychiatry.

[134] Faralli A., Dagna F., Albera A., Bekku Y., Oohashi T., Albera R., Rossi F., Carulli D. (2016). Modifications of perineuronal nets and remodelling of excitatory and inhibitory afferents during vestibular compensation in the adult mouse. Brain Struct. Funct..

[135] Christensen A.C., Lensjø K.K., Lepperød M.E., Dragly S.-A., Sutterud H., Blackstad J.S., Fyhn M., Hafting T. (2021). Perineuronal nets stabilize the grid cell network. Nat. Commun..

[136] Sun Z.Y., Bozzelli P.L., Caccavano A., Allen M., Balmuth J., Vicini S., Wu J., Conant K. (2018). Disruption of perineuronal nets increases the frequency of sharp wave ripple events. Hippocampus.

[137] Orlando C., Ster J., Gerber U., Fawcett J.W., Raineteau O. (2012). Perisynaptic Chondroitin Sulfate Proteoglycans Restrict Structural Plasticity in an Integrin-Dependent Manner. J. Neurosci..

[138] Faini G., Aguirre A., Landi S., Lamers D., Pizzorusso T., Ratto G.M., Deleuze C., Bacci A. (2018). Perineuronal nets control visual input via thalamic recruitment of cortical PV interneurons. eLife.

[139] Al’joboori Y.D., Edgerton V.R., Ichiyama R.M. (2020). Effects of Rehabilitation on Perineural Nets and Synaptic Plasticity Following Spinal Cord Transection. Brain Sci..

[140] Milton A.J., Kwok J.C.F., McClellan J., Randall S.G., Lathia J.D., Warren P.M., Silver D.J., Silver J. (2023). Recovery of Forearm and Fine Digit Function After Chronic Spinal Cord Injury by Simultaneous Blockade of Inhibitory Matrix Chondroitin Sulfate Proteoglycan Production and the Receptor PTPσ. J. Neurotrauma.

[141] Tsien R.Y. (2013). Very long-term memories may be stored in the pattern of holes in the perineuronal net. Proc. Natl. Acad. Sci. USA.

[142] Ramsaran A.I., Wang Y., Golbabaei A., Aleshin S., De Snoo M.L., Yeung B.A., Rashid A.J., Awasthi A., Lau J., Tran L.M. (2023). A shift in the mechanisms controlling hippocampal engram formation during brain maturation. Science.

[143] Huang H., Joffrin A.M., Zhao Y., Miller G.M., Zhang G.C., Oka Y., Hsieh-Wilson L.C. (2023). Chondroitin 4-*O*-sulfation regulates hippocampal perineuronal nets and social memory. Proc. Natl. Acad. Sci. USA.

[144] Burket J.A., Webb J.D., Deutsch S.I. (2021). Perineuronal Nets and Metal Cation Concentrations in the Microenvironments of Fast-Spiking, Parvalbumin-Expressing GABAergic Interneurons: Relevance to Neurodevelopment and Neurodevelopmental Disorders. Biomolecules.

[145] Chang K., Lin L., Cui T., Zhao H., Li J., Liu C., Gao D., Lu S. (2023). Zinc-a2-Glycoprotein Acts as a Component of PNN to Protect Hippocampal Neurons from Apoptosis. Mol. Neurobiol..

[146] Wegrzyn D., Juckel G., Faissner A. (2022). Structural and Functional Deviations of the Hippocampus in Schizophrenia and Schizophrenia Animal Models. Int. J. Mol. Sci..

[147] Miyamae T., Chen K., Lewis D.A., Gonzalez-Burgos G. (2017). Distinct Physiological Maturation of Parvalbumin-Positive Neuron Subtypes in Mouse Prefrontal Cortex. J. Neurosci..

[148] Cho K.K.A., Hoch R., Lee A.T., Patel T., Rubenstein J.L.R., Sohal V.S. (2015). Gamma Rhythms Link Prefrontal Interneuron Dysfunction with Cognitive Inflexibility in Dlx5/6+/− Mice. Neuron.

[149] Maas D.A., Eijsink V.D., Spoelder M., Van Hulten J.A., De Weerd P., Homberg J.R., Vallès A., Nait-Oumesmar B., Martens G.J.M. (2020). Interneuron hypomyelination is associated with cognitive inflexibility in a rat model of schizophrenia. Nat. Commun..

[150] Patrono E., Hrůzova K., Svoboda J., Stuchlík A. (2023). The role of optogenetic stimulations of parvalbumin-positive interneurons in the prefrontal cortex and the ventral hippocampus on an acute MK-801 model of schizophrenia-like cognitive inflexibility. Schizophr. Res..

[151] Kaar S.J., Angelescu I., Marques T.R., Howes O.D. (2019). Pre-frontal parvalbumin interneurons in schizophrenia: A meta-analysis of post-mortem studies. J. Neural Transm..

[152] Zhang W.-J., Shi H.-Z., Guo M.-N., Xu L.-F., Zhai H.-R., Liu Z.-Z., Zhu Y.-Q., Zhang W.-N., Wang J. (2023). PGC-1α regulates critical period onset/closure, mediating cortical plasticity. Front. Mol. Neurosci..

[153] Rankin-Gee E.K., McRae P.A., Baranov E., Rogers S., Wandrey L., Porter B.E. (2015). Perineuronal net degradation in epilepsy. Epilepsia.

[154] Tewari B.P., Chaunsali L., Prim C.E., Sontheimer H. (2022). A glial perspective on the extracellular matrix and perineuronal net remodeling in the central nervous system. Front. Cell. Neurosci..

[155] Crapser J.D., Spangenberg E.E., Barahona R.A., Arreola M.A., Hohsfield L.A., Green K.N. (2020). Microglia facilitate loss of perineuronal nets in the Alzheimer’s disease brain. EBioMedicine.

[156] Wingert J.C., Ramos J.D., Reynolds S.X., Gonzalez A.E., Rose R.M., Hegarty D.M., Aicher S.A., Bailey L.G., Brown T.E., Abbas A.I. (2024). Perineuronal nets in the rat medial prefrontal cortex alter hippocampal-prefrontal oscillations and reshape cocaine self-administration memories. Neuroscience.

[157] Sánchez-Ventura J., Giménez-Llort L., Penas C., Udina E. (2021). Voluntary wheel running preserves lumbar perineuronal nets, enhances motor functions and prevents hyperreflexia after spinal cord injury. Exp. Neurol..

[158] Wang H., Rivenson Y., Jin Y., Wei Z., Gao R., Günaydın H., Bentolila L.A., Kural C., Ozcan A. (2019). Deep learning enables cross-modality super-resolution in fluorescence microscopy. Nat. Methods.

[159] Oláh S., Füle M., Komlósi G., Varga C., Báldi R., Barzó P., Tamás G. (2009). Regulation of cortical microcircuits by unitary GABA-mediated volume transmission. Nature.

[160] Karayannis T., Elfant D., Huerta-Ocampo I., Teki S., Scott R.S., Rusakov D.A., Jones M.V., Capogna M. (2010). Slow GABA Transient and Receptor Desensitization Shape Synaptic Responses Evoked by Hippocampal Neurogliaform Cells. J. Neurosci..

[161] Spoleti E., La Barbera L., Cauzzi E., De Paolis M.L., Saba L., Marino R., Sciamanna G., Di Lazzaro V., Keller F., Nobili A. (2024). Dopamine neuron degeneration in the Ventral Tegmental Area causes hippocampal hyperexcitability in experimental Alzheimer’s Disease. Mol. Psychiatry.

[162] Cardarelli R.A., Martin R., Jaaro-Peled H., Sawa A., Powell E.M., O’Donnell P. (2018). Dominant-Negative DISC1 Alters the Dopaminergic Modulation of Inhibitory Interneurons in the Mouse Prefrontal Cortex. Mol. Neuropsychiatry.

[163] Takiguchi M., Miyashita K., Yamazaki K., Funakoshi K. (2022). Chondroitinase ABC Administration Facilitates Serotonergic Innervation of Motoneurons in Rats with Complete Spinal Cord Transection. Front. Integr. Neurosci..

[164] Dembitskaya Y., Gavrilov N., Kraev I., Doronin M., Tang Y., Li L., Semyanov A. (2021). Attenuation of the extracellular matrix increases the number of synapses but suppresses synaptic plasticity through upregulation of SK channels. Cell Calcium.

[165] Korotchenko S., Zanacchi F.C., Diaspro A., Dityatev A. (2014). Zooming in on the (Peri)synaptic Extracellular Matrix. Neuromethods.

[166] Dzyubenko E., Rozenberg A., Hermann D.M., Faissner A. (2016). Colocalization of synapse marker proteins evaluated by STED-microscopy reveals patterns of neuronal synapse distribution in vitro. J. Neurosci. Methods.

[167] Tewari B., Sontheimer H. (2019). Protocol to Quantitatively Assess the Structural Integrity of Perineuronal Nets ex vivo. BIO-Protoc..

[168] Burket J.A., Urbano M.R., Deutsch S.I. (2017). Sugarcoated Perineuronal Nets Regulate “GABAergic” Transmission: Bittersweet Hypothesis in Autism Spectrum Disorder. Clin. Neuropharmacol..

[169] Berezin V., Walmod P.S., Filippov M., Dityatev A. (2014). Targeting of ECM molecules and their metabolizing enzymes and receptors for the treatment of CNS diseases. Progress in Brain Research.

[170] Yick L.-W., So K.-F., Cheung P.-T., Wu W.-T. (2004). Lithium Chloride Reinforces the Regeneration-Promoting Effect of Chondroitinase ABC on Rubrospinal Neurons after Spinal Cord Injury. J. Neurotrauma.

[171] Eisele B.S., Luka Z., Wu A.J., Yang F., Hale A.T., York J.D. (2021). Sulfation of glycosaminoglycans depends on the catalytic activity of lithium-inhibited phosphatase BPNT2 in vitro. J. Biol. Chem..

[172] Zhao R.-R., Muir E.M., Alves J.N., Rickman H., Allan A.Y., Kwok J.C., Roet K.C.D., Verhaagen J., Schneider B.L., Bensadoun J.-C. (2011). Lentiviral vectors express chondroitinase ABC in cortical projections and promote sprouting of injured corticospinal axons. J. Neurosci. Methods.

[173] Alves J.N., Muir E.M., Andrews M.R., Ward A., Michelmore N., Dasgupta D., Verhaagen J., Moloney E.B., Keynes R.J., Fawcett J.W. (2014). AAV vector-mediated secretion of chondroitinase provides a sensitive tracer for axonal arborisations. J. Neurosci. Methods.

[174] Blanco I., Conant K. (2021). Extracellular matrix remodeling with stress and depression: Studies in human, rodent and zebrafish models. Eur. J. Neurosci..

[175] Paveliev M., Fenrich K.K., Kislin M., Kuja-Panula J., Kulesskiy E., Varjosalo M., Kajander T., Mugantseva E., Ahonen-Bishopp A., Khiroug L. (2016). HB-GAM (pleiotrophin) reverses inhibition of neural regeneration by the CNS extracellular matrix. Sci. Rep..

[176] Rauvala H., Paveliev M., Kuja-Panula J., Kulesskaya N. (2017). Inhibition and enhancement of neural regeneration by chondroitin sulfate proteoglycans. Neural Regen. Res..

[177] Kulesskaya N., Molotkov D., Sliepen S., Mugantseva E., Garcia Horsman A., Paveliev M., Rauvala H. (2021). Heparin-Binding Growth-Associated Molecule (Pleiotrophin) Affects Sensory Signaling and Selected Motor Functions in Mouse Model of Anatomically Incomplete Cervical Spinal Cord Injury. Front. Neurol..

[178] Kulesskaya N., Mugantseva E., Minkeviciene R., Acosta N., Rouhiainen A., Kuja-Panula J., Kislin M., Piirainen S., Paveliev M., Rauvala H. (2022). Low-Molecular Weight Protamine Overcomes Chondroitin Sulfate Inhibition of Neural Regeneration. Front. Cell Dev. Biol..

[179] Alilain W.J., Horn K.P., Hu H., Dick T.E., Silver J. (2011). Functional regeneration of respiratory pathways after spinal cord injury. Nature.

[180] Massey J.M., Hubscher C.H., Wagoner M.R., Decker J.A., Amps J., Silver J., Onifer S.M. (2006). Chondroitinase ABC Digestion of the Perineuronal Net Promotes Functional Collateral Sprouting in the Cuneate Nucleus after Cervical Spinal Cord Injury. J. Neurosci..

[181] Varbanov H., Jia S., Kochlamazashvili G., Bhattacharya S., Buabeid M.A., El Tabbal M., Hayani H., Stoyanov S., Sun W., Thiesler H. (2023). Rescue of synaptic and cognitive functions in polysialic acid-deficient mice and dementia models by short polysialic acid fragments. Neurobiol. Dis..

[182] Wang Y., LeDue J.M., Murphy T.H. (2022). Multiscale imaging informs translational mouse modeling of neurological disease. Neuron.

[183] Paveliev M., Kislin M., Molotkov D., Yuryev M., Rauvala H., Khiroug L. (2014). Acute Brain Trauma in Mice Followed By Longitudinal Two-photon Imaging. J. Vis. Exp..

[184] Sparks F.T., Liao Z., Li W., Grosmark A., Soltesz I., Losonczy A. (2020). Hippocampal adult-born granule cells drive network activity in a mouse model of chronic temporal lobe epilepsy. Nat. Commun..

[185] Fu S., Shi W., Luo T., He Y., Zhou L., Yang J., Yang Z., Liu J., Liu X., Guo Z. (2023). Field-dependent deep learning enables high-throughput whole-cell 3D super-resolution imaging. Nat. Methods.

[186] Peddie C.J., Genoud C., Kreshuk A., Meechan K., Micheva K.D., Narayan K., Pape C., Parton R.G., Schieber N.L., Schwab Y. (2022). Volume electron microscopy. Nat. Rev. Methods Primer.

[187] Eisenstein M. (2023). Seven technologies to watch in 2023. Nature.

[188] Micheva K.D., Gong B., Collman F., Weinberg R.J., Smith S.J., Trimmer J.S., Murray K.D. (2023). Developing a Toolbox of Antibodies Validated for Array Tomography-Based Imaging of Brain Synapses. eNeuro.

[189] Villanueva C.B., Stephensen H.J.T., Mokso R., Benraiss A., Sporring J., Goldman S.A. (2023). Astrocytic engagement of the corticostriatal synaptic cleft is disrupted in a mouse model of Huntington’s disease. Proc. Natl. Acad. Sci. USA.

[190] Tamada H. (2023). Three-dimensional ultrastructure analysis of organelles in injured motor neuron. Anat. Sci. Int..

[191] Haase R., Fazeli E., Legland D., Doube M., Culley S., Belevich I., Jokitalo E., Schorb M., Klemm A., Tischer C. (2022). A Hitchhiker’s guide through the bio-image analysis software universe. FEBS Lett..

[192] Belevich I., Jokitalo E. (2021). DeepMIB: User-friendly and open-source software for training of deep learning network for biological image segmentation. PLoS Comput. Biol..

[193] Hayashi S., Ohno N., Knott G., Molnár Z. (2023). Correlative light and volume electron microscopy to study brain development. Microscopy.

[194] Berg S., Kutra D., Kroeger T., Straehle C.N., Kausler B.X., Haubold C., Schiegg M., Ales J., Beier T., Rudy M. (2019). ilastik: Interactive machine learning for (bio)image analysis. Nat. Methods.

[195] Arzt M., Deschamps J., Schmied C., Pietzsch T., Schmidt D., Tomancak P., Haase R., Jug F. (2022). LABKIT: Labeling and Segmentation Toolkit for Big Image Data. Front. Comput. Sci..

[196] St. Pierre M., Duck S.A., Nazareth M., Fung C., Jantzie L.L., Chavez-Valdez R. (2023). Unbiased Quantitative Single-Cell Morphometric Analysis to Identify Microglia Reactivity in Developmental Brain Injury. Life.

[197] Zhang J., Xie Y., Wu Q., Xia Y. (2019). Medical image classification using synergic deep learning. Med. Image Anal..

[198] Jiang N., Yu F. (2020). A Cell Counting Framework Based on Random Forest and Density Map. Appl. Sci..

[199] Jiang N., Yu F. (2021). A Two-Path Network for Cell Counting. IEEE Access.

[200] Koyuncu C.F., Gunesli G.N., Cetin-Atalay R., Gunduz-Demir C. (2020). DeepDistance: A multi-task deep regression model for cell detection in inverted microscopy images. Med. Image Anal..

[201] Wang S., Lin B., Lin G., Lin R., Huang F., Liu W., Wang X., Liu X., Zhang Y., Wang F. (2020). Automated label-free detection of injured neuron with deep learning by two-photon microscopy. J. Biophotonics.

[202] Cai Y., Zhang X., Kovalsky S.Z., Ghashghaei H.T., Greenbaum A. (2021). Detection and classification of neurons and glial cells in the MADM mouse brain using RetinaNet. PLoS ONE.

[203] Chen D., Nauen D.W., Park H.-C., Li D., Yuan W., Li A., Guan H., Kut C., Chaichana K.L., Bettegowda C. (2021). Label-free imaging of human brain tissue at subcellular resolution for potential rapid intra-operative assessment of glioma surgery. Theranostics.

[204] Axer M., Amunts K. (2022). Scale matters: The nested human connectome. Science.

[205] Traver V.J., Pla F., Miquel M., Carbo-Gas M., Gil-Miravet I., Guarque-Chabrera J. (2019). Cocaine-Induced Preference Conditioning: A Machine Vision Perspective. Neuroinformatics.

[206] Campagner A., Ciucci D., Svensson C.-M., Figge M.T., Cabitza F. (2021). Ground truthing from multi-rater labeling with three-way decision and possibility theory. Inf. Sci..

[207] Isola P., Zhu J.-Y., Zhou T., Efros A.A. (2018). Image-to-Image Translation with Conditional Adversarial Networks 2018. arXiv.

[208] Goodfellow I., Pouget-Abadie J., Mirza M., Xu B., Warde-Farley D., Ozair S., Courville A., Bengio Y. Generative Adversarial Nets. Proceedings of the Advances in Neural Information Processing Systems 27: Annual Conference on Neural Information Processing Systems 2014.

[209] Hinton G.E., Srivastava N., Krizhevsky A., Sutskever I., Salakhutdinov R.R. (2012). Improving neural networks by preventing co-adaptation of feature detectors. arXiv.

[210] Ioffe S., Szegedy C. (2015). Batch Normalization: Accelerating Deep Network Training by Reducing Internal Covariate Shift. arXiv.

[211] Nikolenko S., Kadurin A., Arkhangelskaya E. (2018). Deep Learning.

[212] Krasilnikov N. (2011). Digital Processing of 2D and 3D Images: A Tutorial.

[213] Zeiler M.D., Krishnan D., Taylor G.W., Fergus R. (2010). Deconvolutional networks. Proceedings of the 2010 IEEE Computer Society Conference on Computer Vision and Pattern Recognition.

[214] Ronneberger O., Fischer P., Brox T., Navab N., Hornegger J., Wells W.M., Frangi A.F. (2015). U-Net: Convolutional Networks for Biomedical Image Segmentation. Medical Image Computing and Computer-Assisted Intervention—MICCAI 2015.

