# Supplementary material for: Perineuronal Net Microscopy: From Brain Pathology to Artificial Intelligence
Source: Int J Mol Sci. 2024 Apr 11;25(8):4227. doi: 10.3390/ijms25084227 (PMC11049984; doi:10.3390/ijms25084227)
Supplement: Supplementary file 1 [file ijms-25-04227-s001.zip › ijms-2880762-supplementary.pdf]

Supplementary Information for research article “The perineuronal net microscopy: from brain pathology to artificial intelligence”

Table of Contents

Methods for determining cell boundaries of PNN cells using the Pix2Pix GAN model.......... 2
Method of translating an image into an image with a plotted cell outline............................... 2
The idea behind the method 2
Neural network architecture Pix2Pix 3
Training Dataset 7
Model training 7
Comparative analysis of semi-automatic and automatic methods for encircling cells....... 10
Method for translating an image into a cell outline 13
Conclusion 16
References 16

Methods for determining the PNN mesh contours using the Pix2Pix GAN model

Method of translating an image into an image with a plotted mesh outline

The idea behind the method

Let us present the problem of identifying the boundaries of the PNN meshes as a problem of image-to-image translation, in which it is required to build an algorithm for converting the original images into images, on which the PNN cell boundary is drawn. One of the most common architectures for this problem is the Pix2Pix [207] GAN [208] model. To train it, one needs a paired data set, i.e. pairs of original+annotated images.

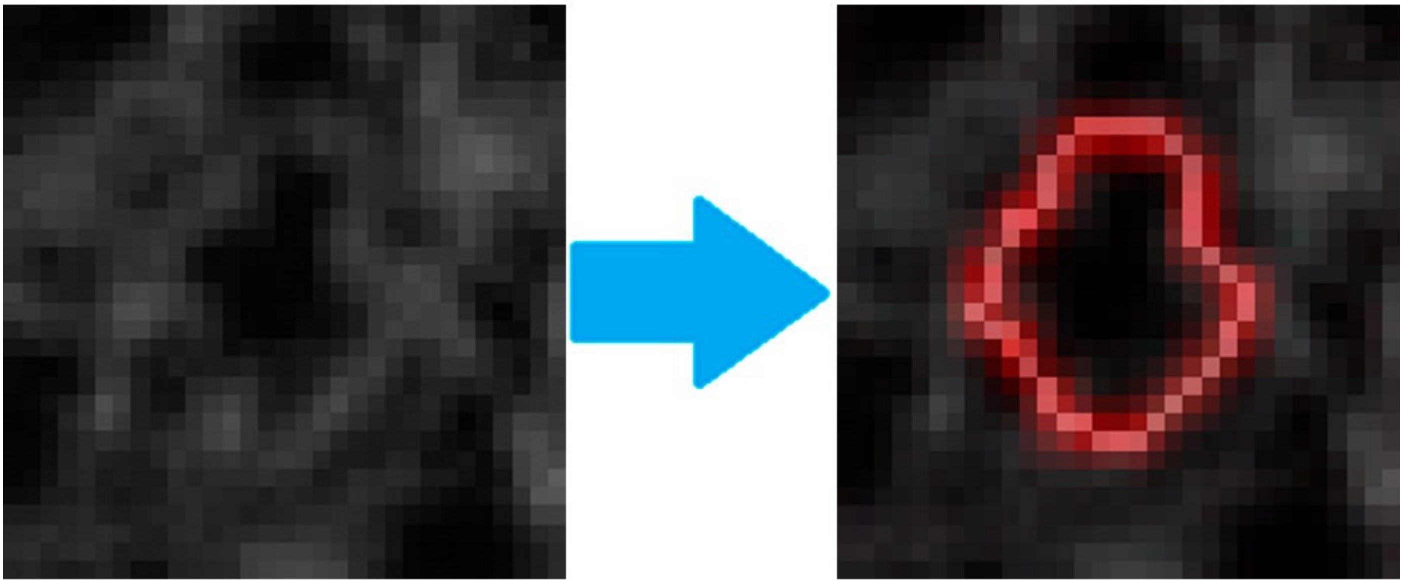

Fig.S1. Extracting the PNN mesh contours as image-to-image translation

Neural network architecture Pix2Pix

Pix2Pix is a deep convolutional architecture designed for image translations. Examples of the Pix2Pix operations can be found in [207].

The generator in Pix2Pix is a convolutional neural network with U-Net-like [214] architecture. In fact, it is an autoencoder, in which the left half acts as an encoder, which translates input image of size $256 \times 256 \times 3$ to the latent vector of size 512, while the right one is a decoder, that transforms the latent vector to the output image of size $256 \times 256 \times 3$. Firstly, 8 convolutional layers with 4×4 kernels eventually produce the latent space vector of 512 size. The convolution layer is the mathematical operation of convolving the input tensor with a kernel (filter), see Eq.S1

$$B_{ij} = (X * K)_{ij} = \sum_{\alpha}^{k-1} \sum_{\beta}^{k-1} X_{i+\alpha,j+\beta} K_{\alpha\beta} \,, 0 \le i,j < x - k + 1 \quad (S1)$$

where X is an input tensor; K is a kernel of size k by k; B is the result of convolution of the X with the kernel K [209]. LeakyReLU activation function is applied to the output of the convolutional layers to add nonlinearity to the model. Unlike sigmoid and hyperbolic functions, the ReLU (Rectified Linear Unit, see Eq.S2) and LeakyReLU (see Eq.S3) activation functions are widely used in modern neural networks, and it is much easier to calculate them [211, p.107]. In our implementation, the encoder nonlinearity is specified using LeakyReLU, and the decoder nonlinearity using ReLU.

$$ReLU(x) = \begin{cases} 0, x < 0, \\ x, x \ge 0 \end{cases} \quad \text{(S2)}$$

$$LeakyReLU(x) = \begin{cases} \alpha x, x < 0, \\ x, x \ge 0 \end{cases} \quad (S3)$$

After the nonlinear activation function, batch normalization is used to average the output tensor values to 0 and bring the standard deviation to 1. Batch normalization or mini-batch data normalization was proposed in [210]. The essence of the method is to normalize mini-batches (tensors) at the outputs of the neural network layers, which helps suppress outliers that arise during the learning process.

The encoder operation result is compression, i.e. reduction of the number of features of the original image. It translates input image with dimensions $256 \times 256 \times 3$ to latent space vector of size 512. In addition, the generator has an important feature: it contains so-called skip connections between layers i and $n - i$, where n is the total number of layers, in our case the skip connections connect 7th layer of encoder with 1st layer of decoder, 6th layer of encoder with 2nd layer of decoder, and so on. The effect of skip connections is carrying information that might have been heavily compressed or disappeared altogether to later layers of the network. After obtaining a layer of minimal dimensions using an encoder, expansion or in other words increase in the number of features using a decoder occurs.

By its structure, the decoder is similar to the encoder, but unlike the latter, instead of convolution, it uses the inverse operation, deconvolution [213], which increases data size. Decoder gets the latent vector of size 512 and then sequentially passes it through deconvolutional layers, producing $256 \times 256 \times 3$ at the end.

Unlike classic GANs, a noise vector is not transmitted to the Pix2Pix generator; all randomness is achieved by receiving Dropout [212] in the generator decoder, which is used not only during training, but also during network operation. In general, Dropout is one of the methods for regularizing models, i.e. combating excessive complexity of models and overtraining. Overtraining is a phenomenon in which a machine learning model overfits the training data and loses its generalizing ability. The idea of Dropout is quite simple: for each neuron in the layer (except for the very last one, i.e. output layer), a certain probability $p = 0.5$ is set with which it will be thrown out of the network (i.e. it will not participate in the calculations) [211, p. 140-141].

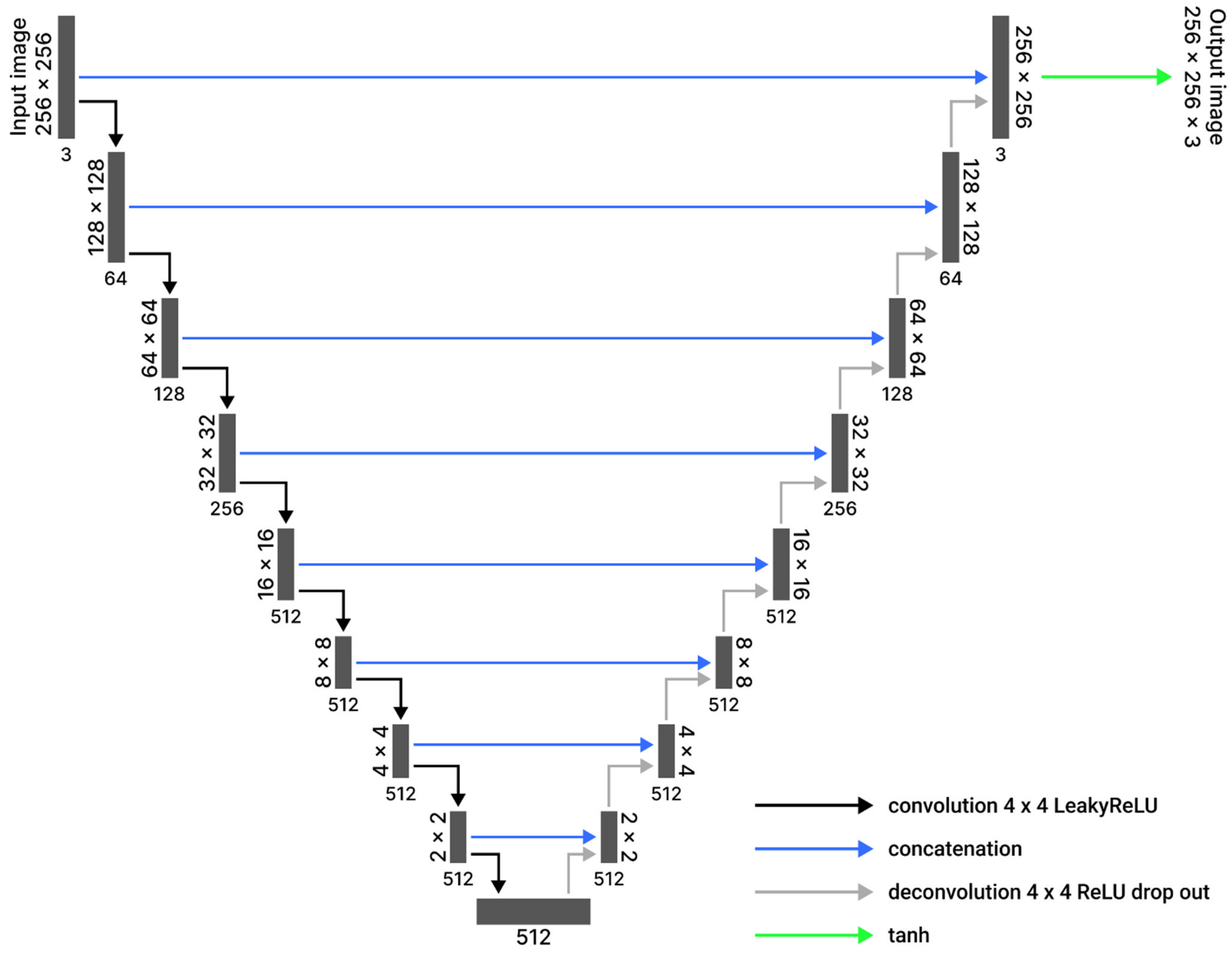

Fig.S2. U-Net architecture used as a generator

The Pix2Pix model uses the PatchGAN [207, p.3] convolutional classifier as a discriminator, which evaluates the reliability of high-frequency information in the image.

The discriminator serves as a complement to the L_1 loss function, which is limited to low frequencies. Below is the formula for the L_1 loss function, where is the result of the model operation and is the true value.

$$L_1 = \sum_{i=1}^{n} \left| y_{true} - y_{predicted} \right|$$

This discriminator works with image fragments, classifying each fragment as real or fake. PatchGAN acts as a style/texture loss function, assuming independence between pixels separated from each other by more than a fragment diameter.

The discriminator is applied to the original-translated image pair, not just to the translated image. To do this, these two images are first combined in depth, i.e. the corresponding image pixels are concatenated. Thus, a tensor with "thick" pixels from 6 channels is obtained and then the discriminator works with this tensor, sequentially applying 5 convolutional layers, 4 of them have batch normalization. LeakyReLU was used as activation function, except for the last layer that uses sigmoid activation. And ultimately obtaining the so-called label map, according to which, using the sigmoidal activation function, a decision is made on whether the image is real or fake.

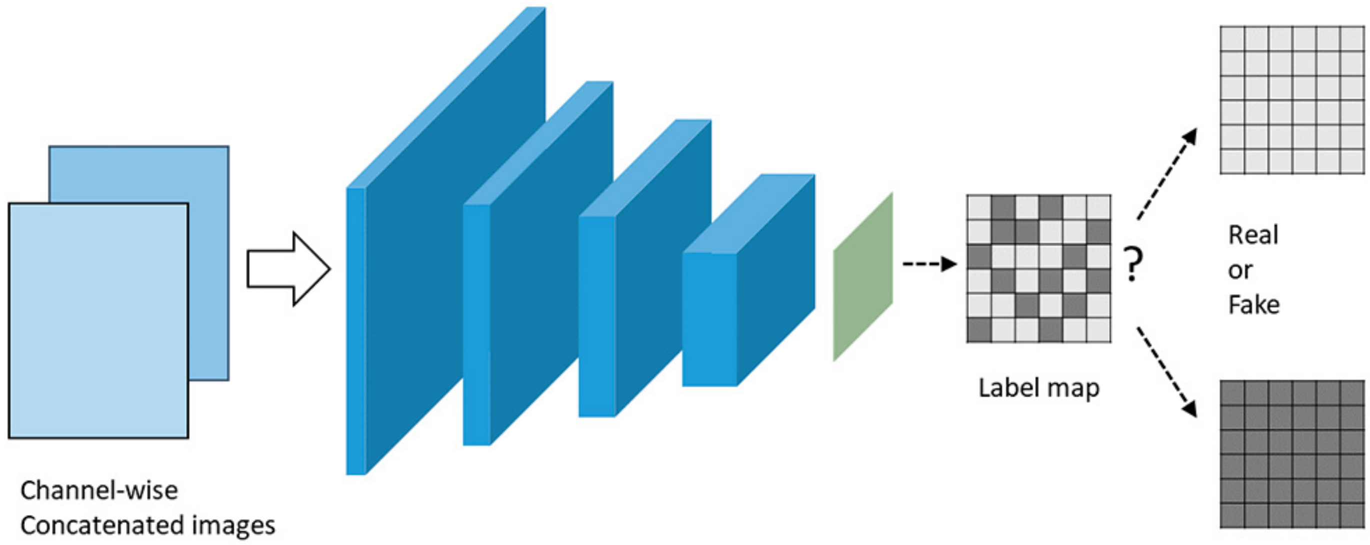

Fig.S3. PatchGAN discriminator architecture

As to the loss functions, the loss functions for the discriminator and generator are calculated using binary cross-entropy, which is calculated as follows:

$$BCELoss(y, \hat{y}) = -\frac{1}{N}\sum_{i=1}^{N}[y_i \log(\hat{y}_i) + (1 - y_i)\log(1 - \hat{y}_i)]$$

where N is the number of elements in the tensors; y_i is the target value for element i; $\hat{y}_i$ is the predicted value for element i.

First, a real pair of images (i.e. before and after marking) is fed into the discriminator, and then an image from the generator is fed into it (producing $D_{output,}$), after that discriminator losses are calculated. The real loss measures how well the discriminator can

distinguish real images, while the fake loss measures its ability to identify fake images. Averaging these two losses helps in training the discriminator to effectively differentiate between real and fake images during the adversarial training process.

$$\mathrm{L}_{\mathrm{D}_{\mathrm{real}}} = \mathrm{B}CELoss(D_{output,}, 1)$$

$$\mathrm{L}_{\mathrm{D}_{\mathrm{fake}}} = \mathrm{B}CELoss(D_{output,}, 0)$$

$$L_D = (L_{D_{real}} + L_{D_{fake}})$$

Then generator loss is calculated, it consists of two parts. First part measures how well the model can distinguish real images

$$L_{cGAN} = \mathrm{B}CELoss(D_{output,}, 1)$$

$$L_{cGAN}(G, D) + \lambda\mathcal{L}_{L1}(G)$$

the second component is the already mentioned L1, weighted by a coefficient $\lambda = 100$.

Training Dataset

A new paired dataset containing 7897 images before and after annotation is used. The outline of a PNN mesh is highlighted in red, making it both visually more noticeable and easily distinguishable from a black and white image. In the dataset, approximately 83% were examples obtained by the semi-automatic algorithm, while the remaining ones were annotated manually (both data sets from [35]).

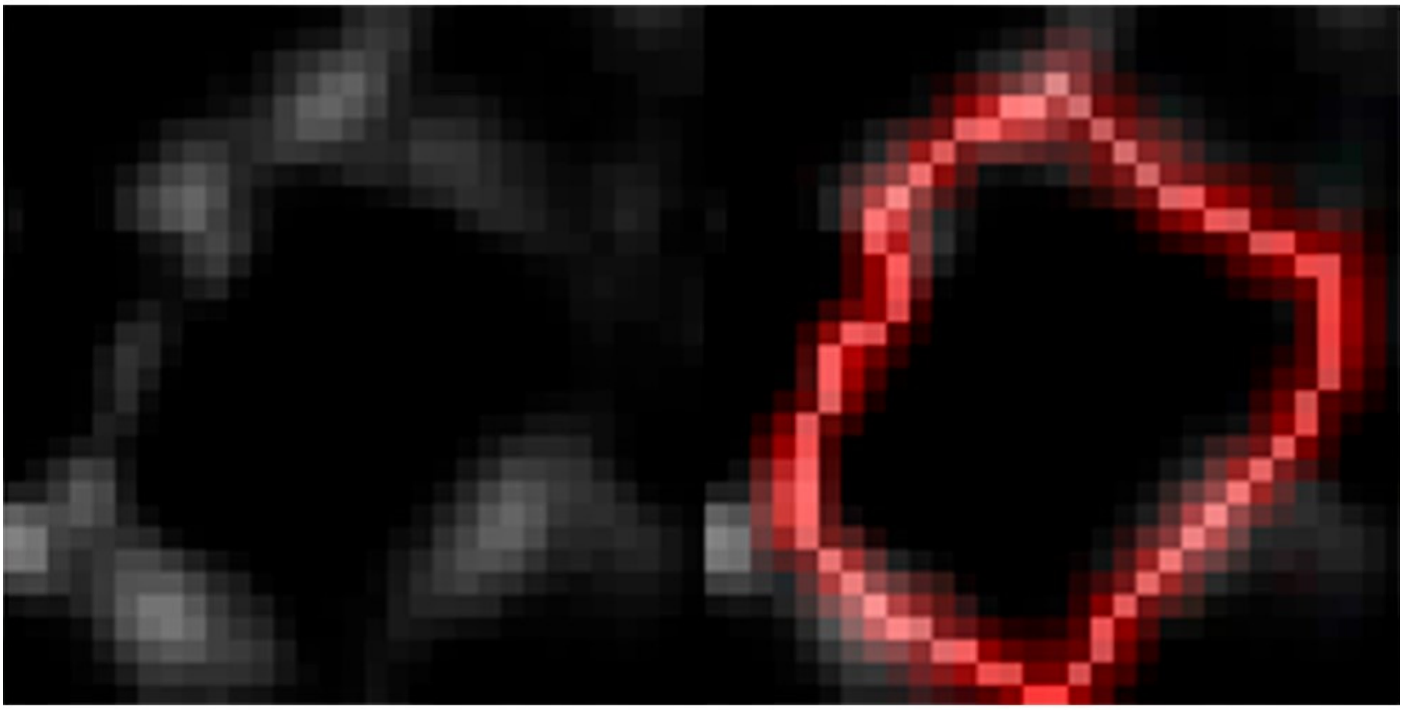

Fig. S4. Example of an image from the dataset

Model training

The Pix2Pix model was implemented on the PyTorch framework and the model was trained on this dataset. Before training, the original dataset was randomly split into two subsets: 80% of the data for training and 20% for testing. The neural network was trained for 155 epochs, with a batch size of 64. Training took about 5 hours on NVIDIA Tesla P100 GPU in Kaggle cloud platform. In Fig. S5 there are the results of the model running for five random test cases during training. One can see how, throughout training, the model learns better and better to determine the cell boundaries.

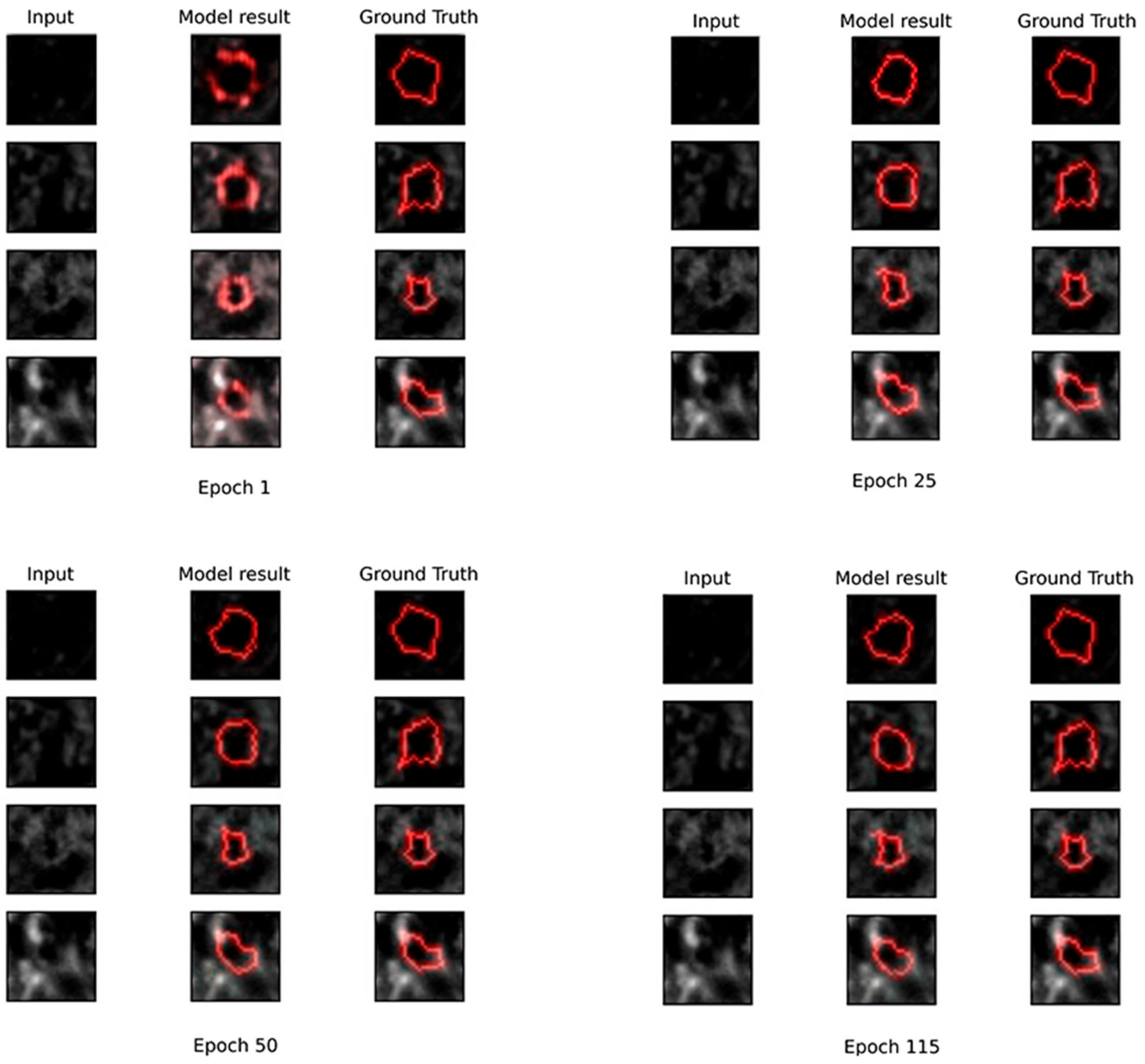

Fig. S5. Results of the model during the learning process

Fig. S6 shows a graph of the training process of the Pix2Pix model. The abscissa axis contains the numbers of training epochs (an epoch is the cycle of complete passage of all training data through the model), whereas the ordinate axis shows the values of the model's loss functions on the training set. The blue line is for losses of the Pix2Pix generator on the training set, while the yellow line shows the discriminator losses.

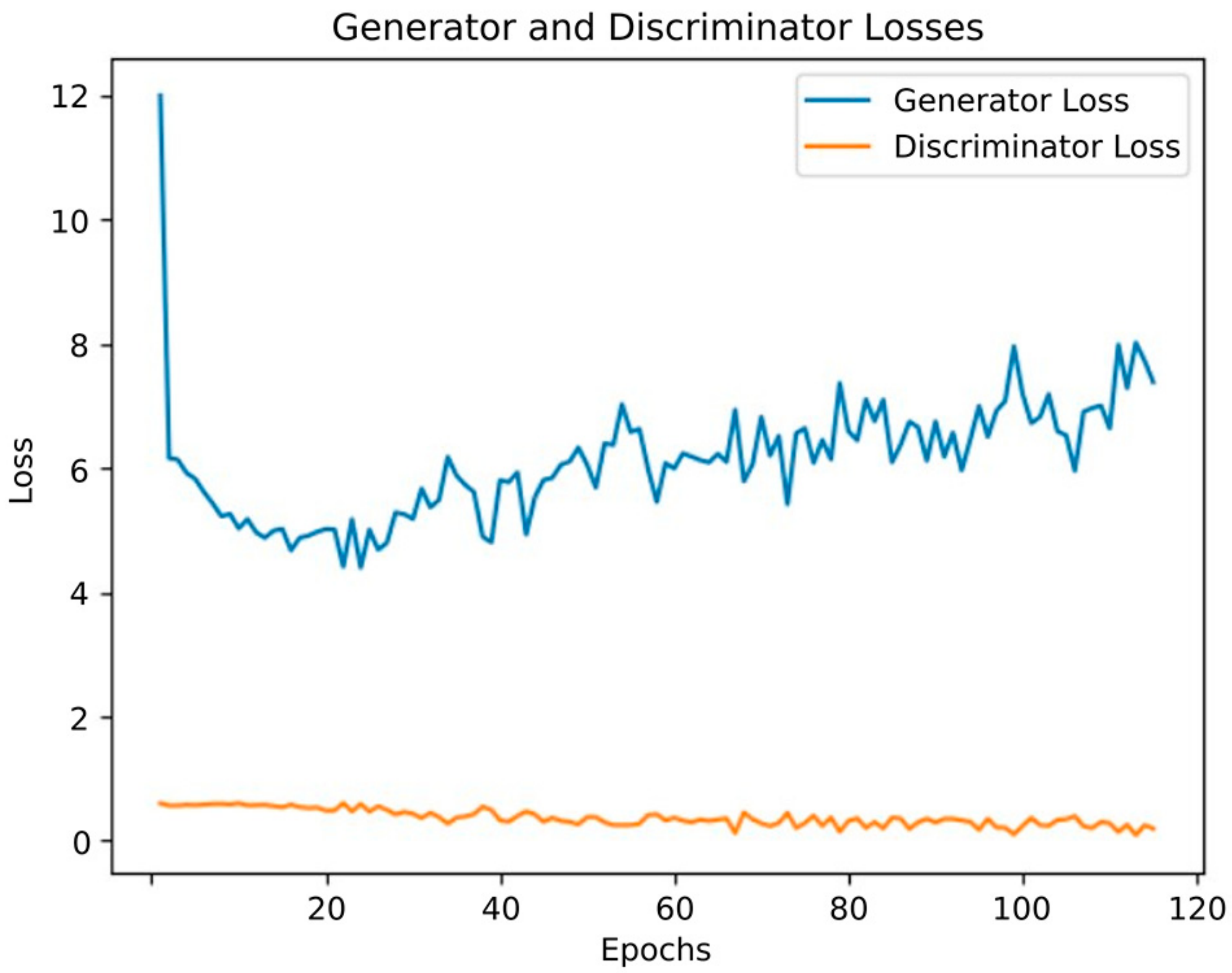

Fig. S6. Graphs depicting losses throughout model training

As can be seen from the graphs, until approximately the 25th epoch the generator losses are dropping rapidly, which is consistent with the above intermediate results during the training process indicating that the generated images are improving at a fast rate. Moving further along the graph, jumps in generator losses and a slowdown in the decline in discriminator losses are observed. Judging by subsequent intermediate results, the quality of the generated images neither improve significantly, nor deteriorate. Fig. S7 shows the results of the trained model operation on 4 random images from the test set.

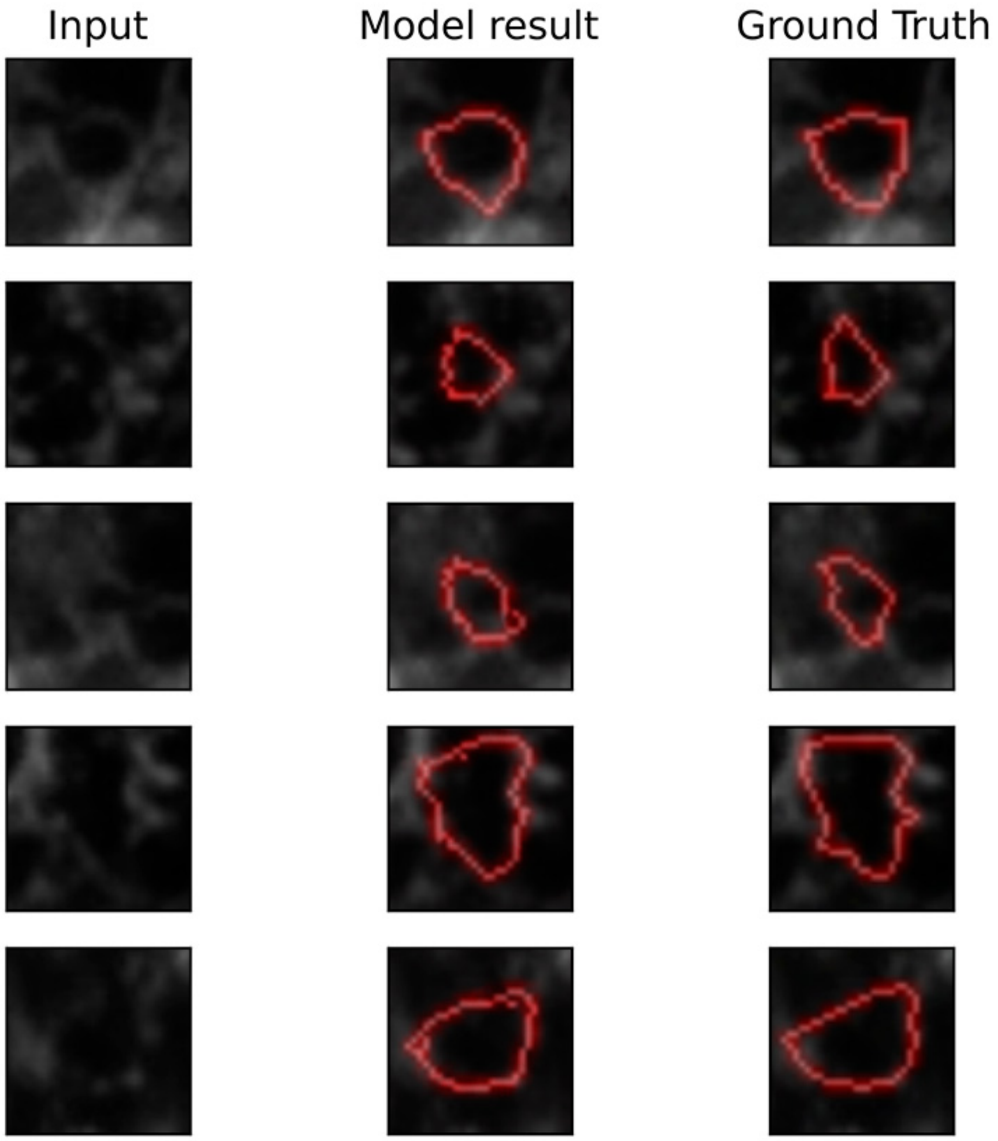

Fig. S7. Results of the trained model

Comparative analysis of semi-automatic and automatic methods for encircling cells

Let us compare various metrics for the results of semi-automatic (the vast majority of examples from the dataset are obtained using it) and automatic (Pix2Pix) methods for encircling cells.

We construct boxplot graphs of the areas and perimeters (Fig. S8) of cells (in pixels) obtained by two methods. Separate circles on the graphs are outliers. From the graphs, one can see that the average values of areas and perimeters obtained by the two methods are quite close.

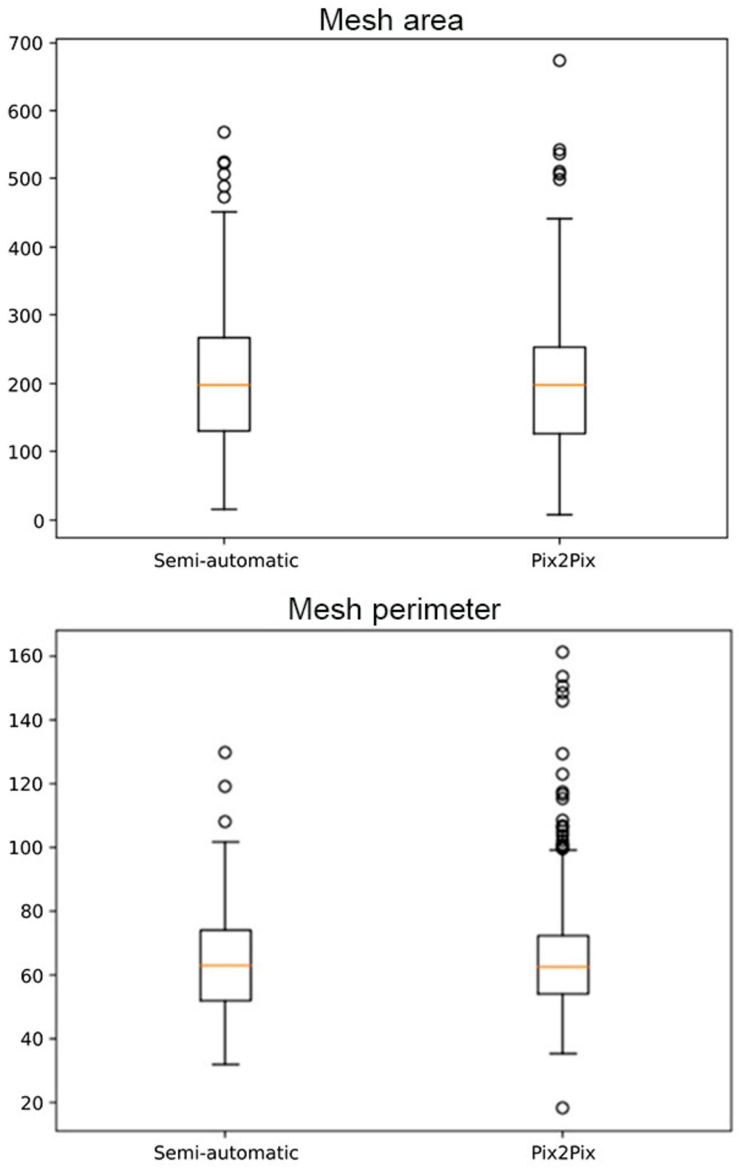

Fig. S8. Boxplot-graph of cell areas and perimeters obtained by two methods

The Fig. S9 shows the correlations of cell metrics obtained by the two methods. The black straight line is linear regression; the red dotted line is a line from the origin at 45 degrees serving as a reference line; R is the Pearson correlation coefficient; Sp is the Spearman correlation coefficient (more resistant to outliers than the Pearson correlation coefficient).

The Pearson correlation coefficient is calculated as follows:

$$\rho = \frac{\sum(x_i - \bar{x})(y_i - \bar{y})}{\sqrt{\sum(x_i - \bar{x})^2 \sum(y_i - \bar{y})^2}}$$

The Spearman correlation coefficient is calculated as follows:

$$r_S = \rho_{R(X)R(Y)} = \frac{Cov(R(X), R(Y))}{\sigma_{R(X)}\sigma_{R(Y)}}$$

where $R(x)$ is the rank, i.e. the ordinal number of the element in the sorted sequence.

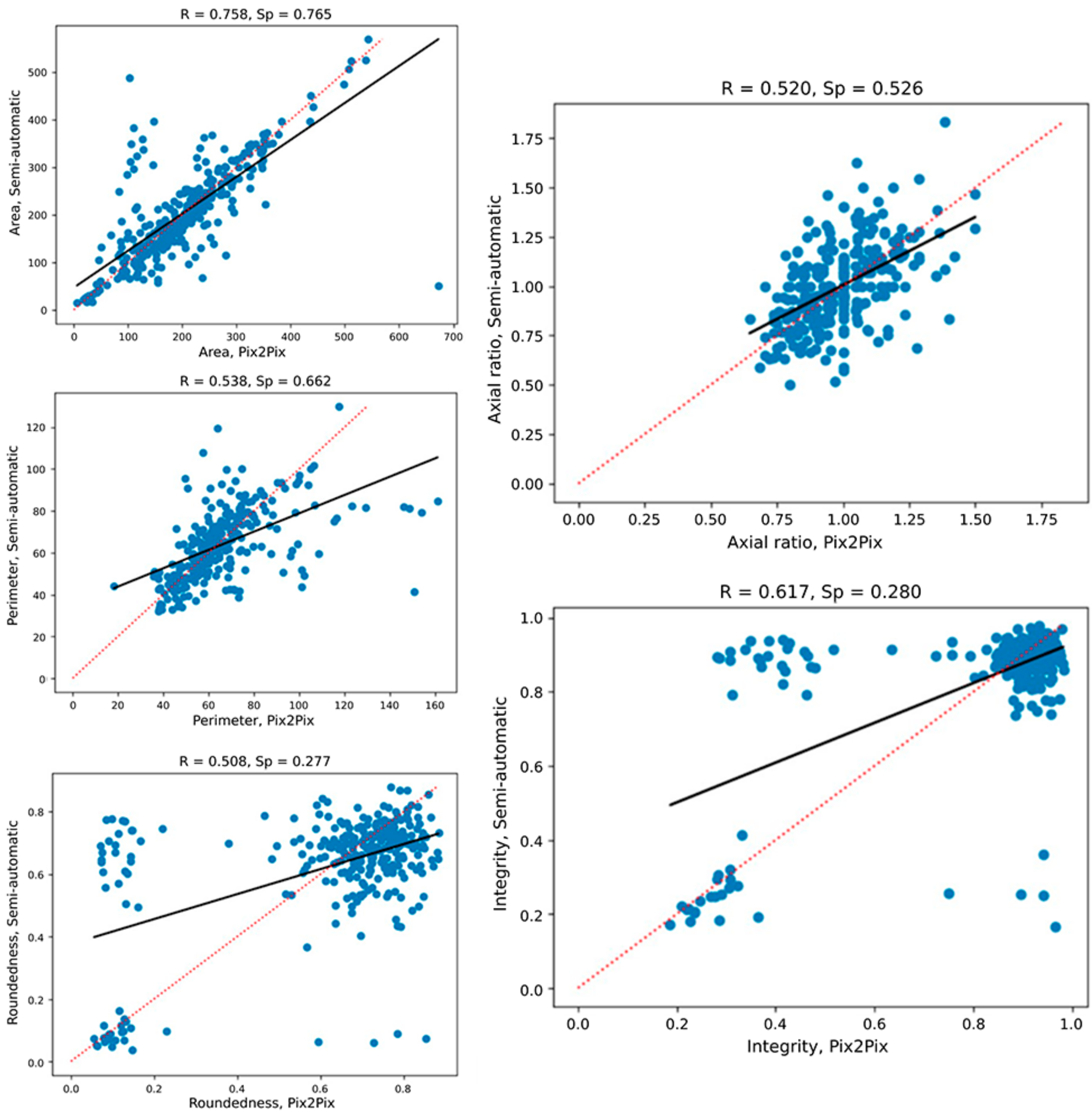

Fig. S9. Correlation of the mesh metrics identified by two methods

Method for translating image-to-contour

The idea behind the method is to translate the source image into a separate PNN mesh contour, rather than drawing the contour in the source image, as in the previous method. Working with a separate cell outline would be much easier than selecting the “finished complete” outline.

The dataset for this method is obtained from the previous dataset as follows: in images with encircled cells, white pixels are painted blue, whereas the remaining pixels are painted white. The result is a set of 173 images. Fig. S10 shows an example from the dataset: original image and the outer boundary of the cell, selected by the semi-automatic algorithm

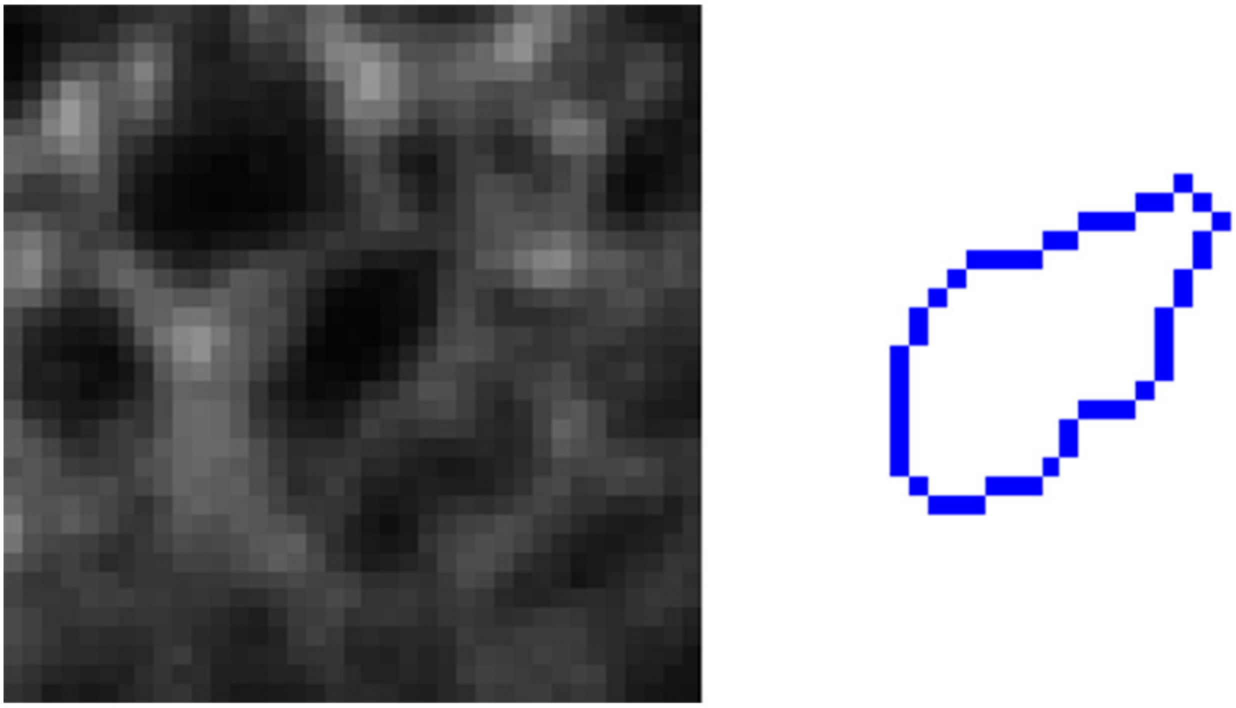

Fig. S10. An example from the dataset

Next, the same Pix2Pix model is trained for 400 epochs with a batch size of 4. The batch size is selected empirically. For example, by setting the batch size to 16, the neural network always produces an empty white image; therefore, for this model and dataset, a relatively small size of the batch is needed. Fig. S11 shows the results of the neural network operation as it progresses through training epochs: As can be seen from the figure above, the neural network reproduces exactly the same shape every time, sometimes adding small artifacts. This indicates that the neural network has not learned to encircle the PNN cells. The latter means that the shape what the neural network reproduces all the time gives a minimal loss function, but does not reflect any dependence on the shape and location of the PNN cell.

The Fig. S11 shows that the generator and discriminator losses stop falling or growing, and they only fluctuate over more than 200 epochs. This confirms the lack of progress in learning. Thus, undertraining occurred. Considering that the previous method with “additional drawing” of boundaries of the PNN cells is trained quite successfully on the existing data, we can conclude that the Pix2Pix architecture is not suitable for translating images into individual contours of objects.

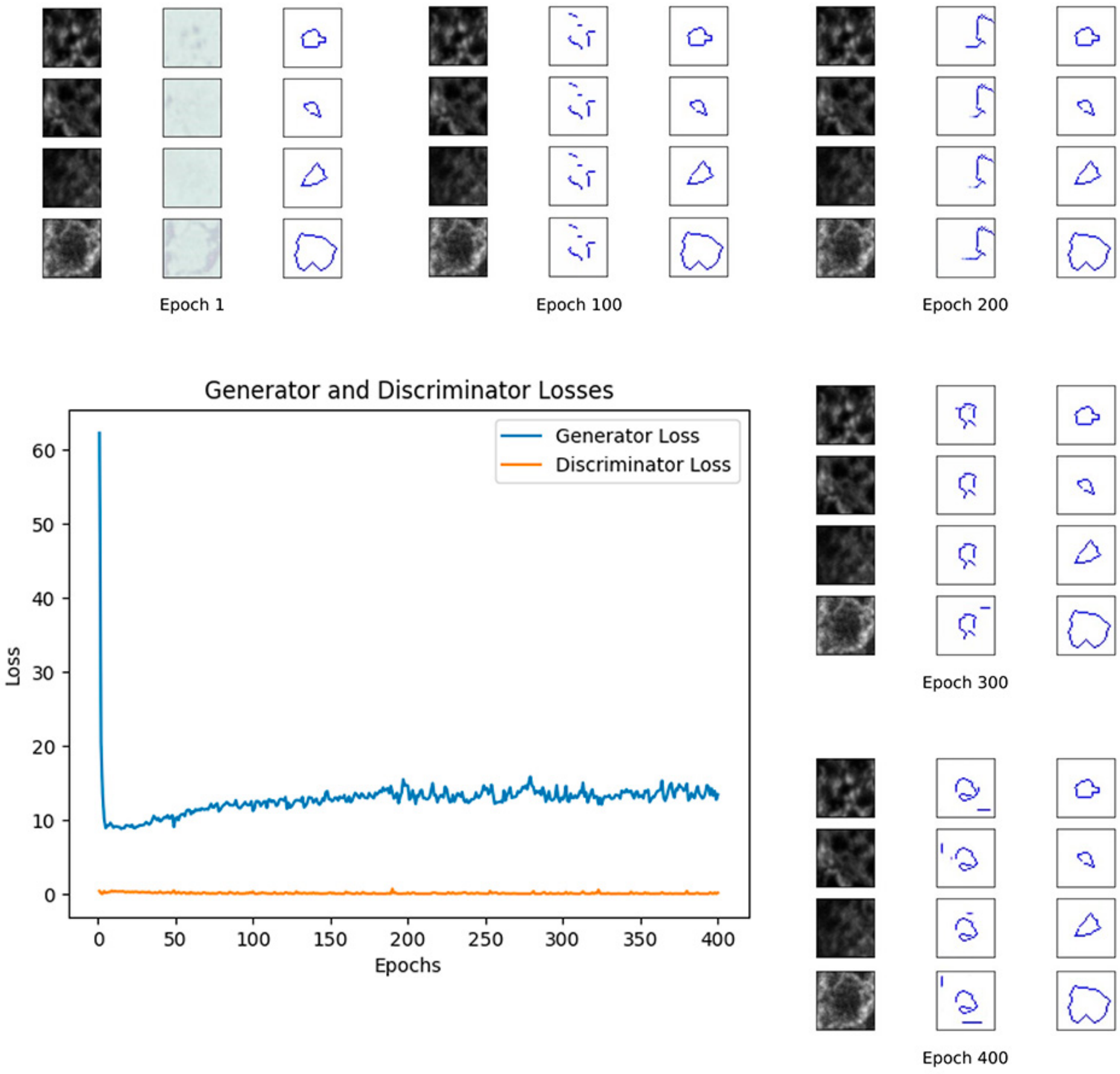

Fig. S11. Results of the model operation during training and loss function plot
